# Temporal network analysis identifies early physiological and transcriptomic indicators of mild drought in *Brassica rapa*

**DOI:** 10.7554/eLife.29655

**Published:** 2017-08-18

**Authors:** Kathleen Greenham, Carmela Rosaria Guadagno, Malia A Gehan, Todd C Mockler, Cynthia Weinig, Brent E Ewers, C Robertson McClung

**Affiliations:** 1 Department of Biological SciencesDartmouth CollegeHanoverUnited States; 2 Department of BotanyUniversity of WyomingLaramieUnited States; 3 Donald Danforth Plant Science CenterSt. LouisUnited States; 4 Department of Molecular BiologyUniversity of WyomingLaramieUnited States; 5 Program in EcologyUniversity of WyomingLaramieUnited States; University of British ColumbiaCanada

**Keywords:** *Brassica rapa*, drought, transcriptomic network analysis, abiotic stress, photosynthesis, daily rhythms, Other

## Abstract

The dynamics of local climates make development of agricultural strategies challenging. Yield improvement has progressed slowly, especially in drought-prone regions where annual crop production suffers from episodic aridity. Underlying drought responses are circadian and diel control of gene expression that regulate daily variations in metabolic and physiological pathways. To identify transcriptomic changes that occur in the crop *Brassica rapa* during initial perception of drought, we applied a co-expression network approach to associate rhythmic gene expression changes with physiological responses. Coupled analysis of transcriptome and physiological parameters over a two-day time course in control and drought-stressed plants provided temporal resolution necessary for correlation of network modules with dynamic changes in stomatal conductance, photosynthetic rate, and photosystem II efficiency. This approach enabled the identification of drought-responsive genes based on their differential rhythmic expression profiles in well-watered versus droughted networks and provided new insights into the dynamic physiological changes that occur during drought.

## eLife digest

Around 60% of the food produced worldwide relies entirely on rain for its water supply. However, in the decades ahead global climate change is predicted to cause droughts to happen more often and become more severe in many regions. Therefore, in order to sustain our food supply we need to better understand how plants respond to drought and then use that knowledge to improve the ability of crops to cope with it.

Unlike animals, plants cannot move away from drought or other stressful situations so they must face these difficulties ‘head on’. For example, when water is in short supply, plants close pores known as stomata on the surface of their leaves to reduce water loss. However, these pores need to be open to allow carbon dioxide gas, which plants use to make sugars in a process called photosynthesis, to enter the plant. Their response to drought must therefore be carefully controlled to make sure that the plant is still capable of performing photosynthesis.

Turnip, napa cabbage, bok choy and field mustard are all varieties of a crop species known as *Brassica rapa.* These crops are grown in relatively dry regions such as the Canadian prairies and northern China, making drought stress a major threat to production.

Previous studies had shown that drought stress causes changes in the activities of genes at certain times of day. To investigate this further, Greenham, Guadagno et al. studied how young *B. rapa* plants grown in a controlled environment with a steady supply of water responded when watering stopped. The experiments show that, even before the plants show obvious signs of drought stress such as wilting, there are extensive changes in the activity of many genes and processes inside plant cells that vary according to the time of day.

Greenham, Guadagno et al. used an analysis technique to bring together all of the data into a network based on similar patterns of changes over time. This identified groups of genes whose changes in activity match the timing of the observed changes in the opening and closing of stomata, photosynthesis and other processes. These represent very early responses to drought stress in the plant.

This work emphasizes the importance of time of day on plant stress responses. Changes that occurred only in the morning could not have been detected by measurements taken in the afternoon, and vice versa. The next step is to find out which of the changes observed in this work are most important in making plants resistant to drought. In the future, these findings may help researchers to develop strategies that would improve drought resistance in crop plants.

## Introduction

Projected impacts of climate change on crop yields vary widely depending on crop type and location; however, rising temperatures, with attendant increases in drought as well as insect and disease outbreaks, are predicted to result in net losses in yield of North American crops by the end of the 21st century (Settele et al., 2014). Water stress accounts for the largest proportion of crop loss in the U.S., and an estimated 45% of U.S. land surface suffers from low water availability (DeLucia et al., 2014). To mitigate the predicted increase in water stress on plants (Blum, 2005; Jones and Corlett, 1992; Anderegg et al., 2012) and achieve maximal crop yield potential, locally adapted stress tolerance traits are needed.

In response to soil water deficit, plants can exhibit either drought escape or drought resistance mechanisms (Levitt, 1980; Harb et al., 2010). Under drought escape, plants complete their life cycle before the onset of stress. Drought resistance can occur through dehydration avoidance or through tolerance (Levitt, 1980). With dehydration avoidance, plants maintain high cellular water potential by lowering stomatal conductance and/or reducing water loss through changes in leaf area or orientation and by increasing resource allocation to roots. Drought tolerant plants conserve cell turgor through osmotic adjustments to survive the drought stress (Levitt, 1980) and may also tolerate lower cell water potentials through anisohydric water potential regulation (Franks et al., 2007) while maintaining cellular metabolism (Sade et al., 2012). Depending on the plant species and genotype, a combination of avoidance and tolerance traits may be utilized (Chaves and Oliveira, 2004). The potential allocation changes in drought-stressed plants also depend on selection by herbivores and the true cost of defensive molecules as leaf carbon fixation is reduced by drought (Züst and Agrawal, 2017; Hamilton et al., 2001). Achieving maximum yield while breeding for drought stress responses will likely rely on a balance between avoidance and tolerance strategies (Tuberosa, 2012).

A commonly used measure for assessing drought resistance is the intrinsic water use efficiency (WUE), which is defined as the ratio between stomatal conductance (*g*_s_) and CO_2_ assimilation (*A*) (Condon et al., 2002). WUE is often used as a proxy for drought resistance, but it is not always an accurate predictor of yield capacity under drought (Medrano et al., 2012) especially when biomass allocation to roots increases in response to drought (Edwards et al., 2016) or when yield is tightly correlated with water use (Blum, 2009). Smaller plants that limit water use and have moderate growth or short growing seasons often have higher WUE but low yield potential (Blum, 2005). This argues for using the individual *g*_s_ and *A* measures to separately assess the impact of CO_2_ supply and demand effects on yield.

Studies in numerous plant species have explored transcript level changes following various degrees of drought stress (Zhang et al., 2012; Yamaguchi-Shinozaki and Shinozaki, 2006; Smita et al., 2013; Seki et al., 2002; Shinozaki and Yamaguchi-Shinozaki, 2007; Degenkolbe et al., 2009) and the plant responses to drought at physiological and molecular levels (Sakuma et al., 2006; Yu et al., 2008; Ning et al., 2010). Few studies have evaluated physiological and molecular responses in plants experiencing mild drought (Watkinson et al., 2003; Vásquez-Robinet et al., 2010), although mild drought is more relevant to intensive agricultural settings than severe drought. Many drought-responsive genes are under circadian regulation (Covington et al., 2008) resulting in specific time-of-day responses to drought (Wilkins et al., 2010; Dubois et al., 2017). To associate the relevant transcriptomic changes with physiology, these time-of-day effects must be considered. Temporal changes complicate the assessment of differential gene expression in response to an abiotic stress due to the differences in the phasing of maximal and minimal expression levels for transcripts under circadian or diel control (Greenham and McClung, 2015). Because time-of-day changes in transcript phasing are dominant relative to the responses to drought status, comparisons of gene expression levels at any single time point will chiefly capture time-of-day expression differences rather than drought responses.

Network analysis is a powerful way to extract meaningful differences across treatments, development, or time by providing pathway structure (Priest et al., 2014; Gehan et al., 2015; Righetti et al., 2015). In addition, a network approach facilitates the integration of multiple datasets that can provide support to the network structure and can be used to generate predictive regulatory networks (Langfelder et al., 2011; Krouk et al., 2013). Here, we applied a co-expression network approach to analyze both transcriptome and physiological parameters over a two-day time course in drought-stressed and control plants, providing temporal resolution necessary for correlation of network modules with dynamic changes in drought response.

We performed these studies in the crop species *Brassica rapa*. The genus *Brassica* includes the closest crop relatives of *Arabidopsis* and therefore is an excellent crop model for comparative studies, including analyses of adaptive drought responses. There is tremendous morphological diversity within *Brassica* species with important vegetable, oilseed, and forage crops that have acquired a range of stress response traits (Ashraf and Mehmood, 1990). Rapeseed (*B. napus*), an allopolyploid derived via hybridization between *B. rapa* and *B. oleracea*, is the second largest oil crop after soybean with an annual global production of 70 million tons (http://faostat.fao.org, 2014). The majority of *Brassica* crops are grown in arid and semi-arid regions making drought stress a major determinant of yield. *B. rapa* shows a wide spectrum of drought responses (Yarkhunova et al., 2016; Edwards et al., 2012), suggesting that there is extensive genetic variation to explore. Further, quantitative genetic analyses of *B. rapa* under well-watered and drought conditions found opposite correlations between WUE and shoot biomass: plants with low WUE had higher biomass under well-watered conditions, whereas those with high WUE were larger under drought conditions (Edwards et al., 2012; El-Soda et al., 2014). Subsequent studies revealed quantitative trait locus (QTL) allele contributions to the association between *g*_s_ and total biomass (Edwards et al., 2016).

Here, our objective was to associate the earliest transcriptomic responses to a water deficit with the dynamic changes in physiology throughout the day. To clarify the gene reprogramming under mild drought we focused our attention on the early response to drought stress. We measured several physiological traits and transcript abundances in *B. rapa* over 48 hr of controlled mild drought. To identify important regulatory pathways contributing to drought responses we applied a circadian guided co-expression network approach to correlate changes in temporally regulated transcripts with photosynthetic rate (*A*), stomatal conductance (*g*_s_; a measure of CO_2_ supply), and maximum efficiency of photosystem II (PSII) in light conditions (*Fv’/Fm’*; a measure of available energy for CO_2_ demand). Gene reprogramming was altered over the time course of drought treatment, and significant changes in temporal dynamics of *g*_s_ and *Fv’/Fm’* reveal them to be reliable indicators of early drought perception.

## Results and discussion

### Establishing the mild drought treatment

**Figure 1.**
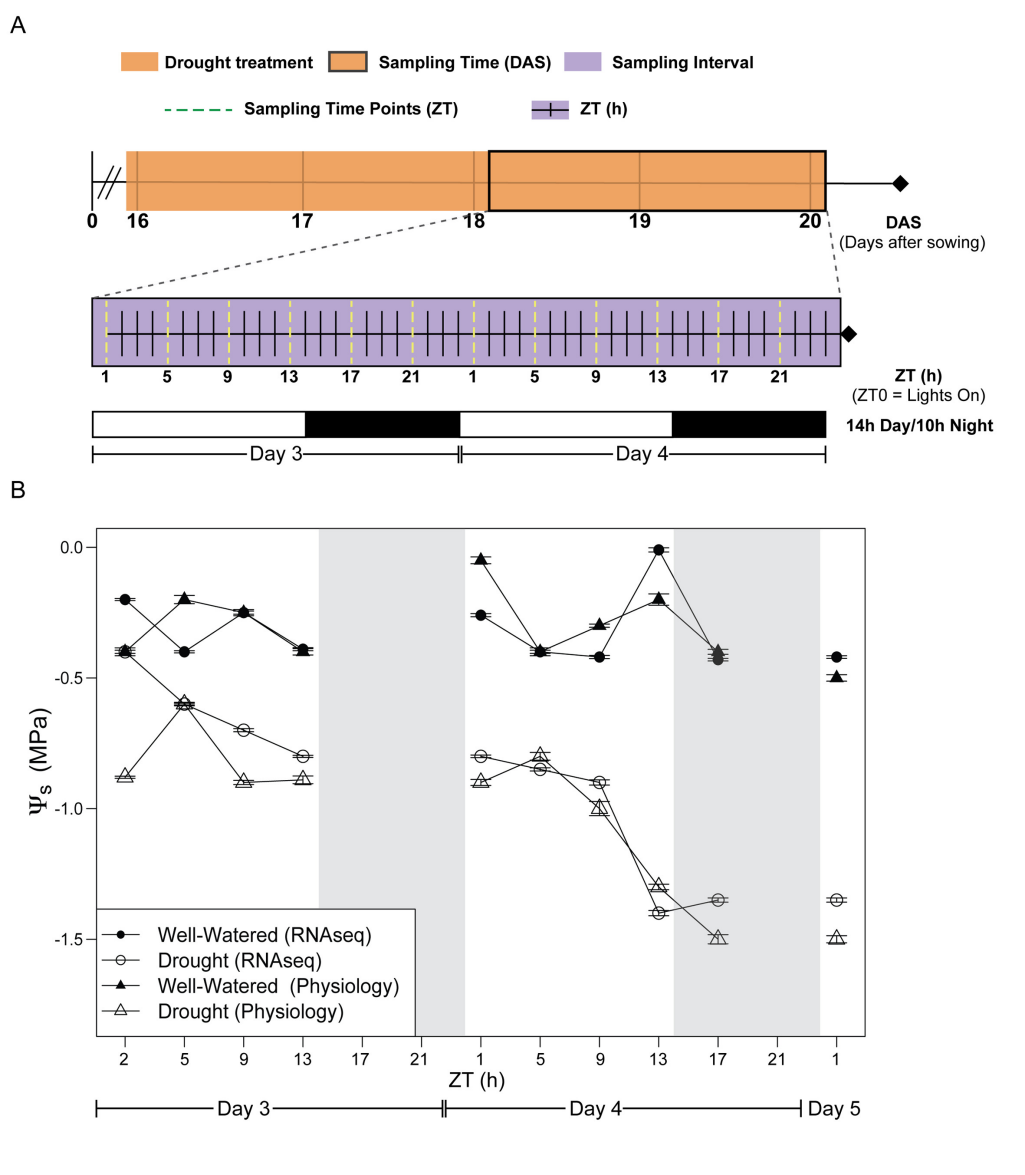
Experimental design for evaluation of early effects of mild drought treatment on *Brassica rapa* R500 plants and soil water potential during the experiment. (**A**) Schematic illustration of the experimental design with days of the experiment and collection/measurement times. Top: The time of the experiment in days after sowing (DAS) is plotted with the orange shade representing the days of mild drought treatment starting at the end of 15 DAS. Center: Purple shade (between 18 and 20 DAS) indicates the 2 days of continuous collection/measurements. The x-axis represents the 48 hr of the experiment in Zeitgeber Time (ZT0 = lights on in the growth chambers). The dotted yellow lines represent the ZT times for collection and measurements of both well-watered and droughted plants. Bottom: White and black bars represent the photoperiod in the growth chambers (14 hr/10 hr; Day/Night) for Days 3 and 4. (**B**) Soil water potential (*Ψ*_s_) progression for well-watered (solid symbols) and mild droughted (open symbols) *B. rapa* R500 plants (n = at least 6) after the start of the drought treatment during the physiology (triangles) and RNA-seq (circles) time-course experiments. 10.7554/eLife.29655.004Figure 1—source data 1.Growth conditions and reproducibility of the mild drought treatment.

**Figure 2.**
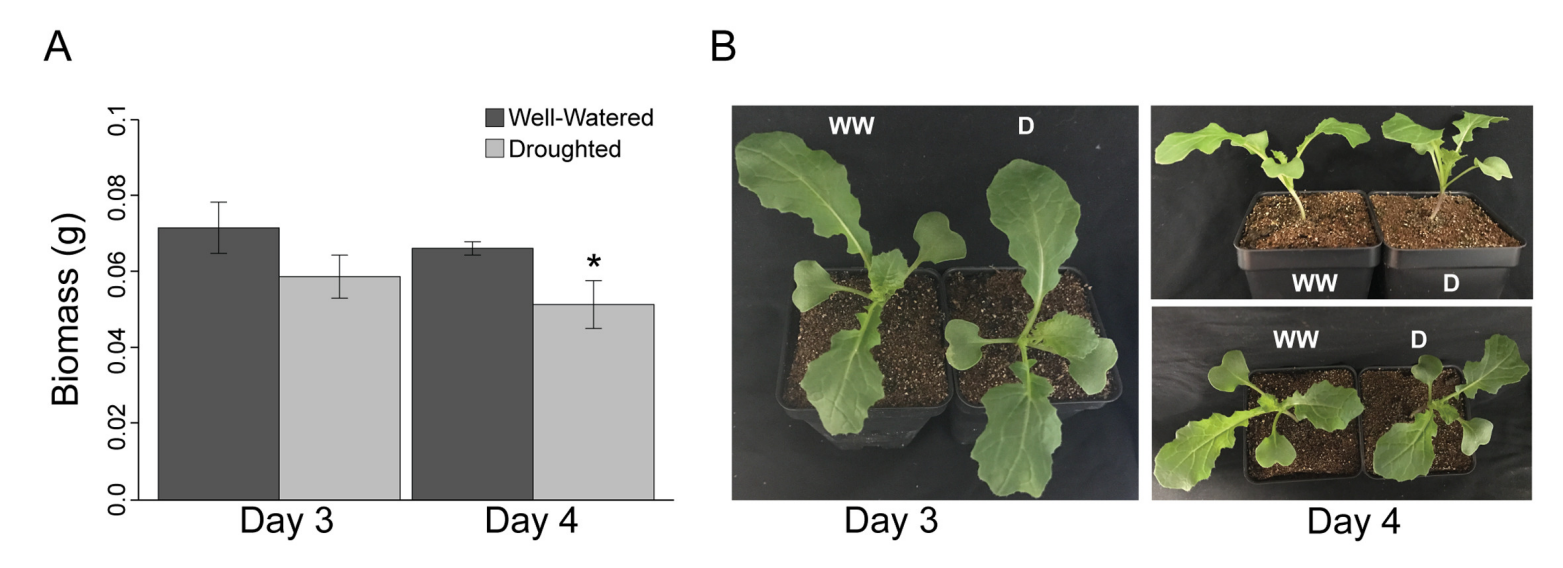
Despite a significant (p<0.001) decrease in dry biomass accumulation, *Brassica rapa* R500 plants did not reach the wilting point in mild drought. (**A**) Grams of dry above-ground biomass in well-watered (dark gray) and mild droughted (light gray) *B. rapa* R500 plants (*n* = 12) collected on Day 3 and Day 4. On both days, above-ground biomass was collected at ZT9. (**B**) Top and lateral view of well-watered and droughted *B. rapa* R500 plants on Day 3 and Day 4 of the experiment.

We assessed the early stages of mild drought, completely withholding water for the droughted cohort of *B. rapa* (Yellow Sarson) R500 beginning at 16 days after sowing (DAS). Tissue sampling and physiological measurements were conducted on Day 3 and 4 of drought, 18 and 19 DAS, respectively (Figure 1A, Figure 1—source data 1). The experiment was performed twice under similar temperature, photoperiod, and soil moisture conditions (Figure 1A, Figure 1—source data 1). In order to assess the reproducibility of our conditions, *Fv’/Fm’* and above-ground biomass were monitored in both experiments, and there were no significant differences between the temporal replicates. After four days of drought, soil water potential (*Ψ*_s_) had declined progressively to −1.5 MPa, whereas *Ψ*_s_ was relatively constant between 0 and −0.5 MPa for the well-watered soil (Figure 1B, Figure 1—source data 1). The droughted plants showed a significant decrease in dry above-ground biomass by the end of Day 4 (Figure 2A); however, there was no wilting during the experiment (Figure 2B). In a previous experiment, prolonged progressive drought resulted in *Ψ*_s_ equal to approximately −5 MPa, yet some R500 plants were able to recover upon re-watering and maintained their gas exchange (Guadagno et al., 2017). Therefore, the drought conditions applied in this study capture the early perception of drought stress (Harb et al., 2010).

### Gas-exchange and chlorophyll *a* fluorescence changes in response to drought

**Figure 3.**
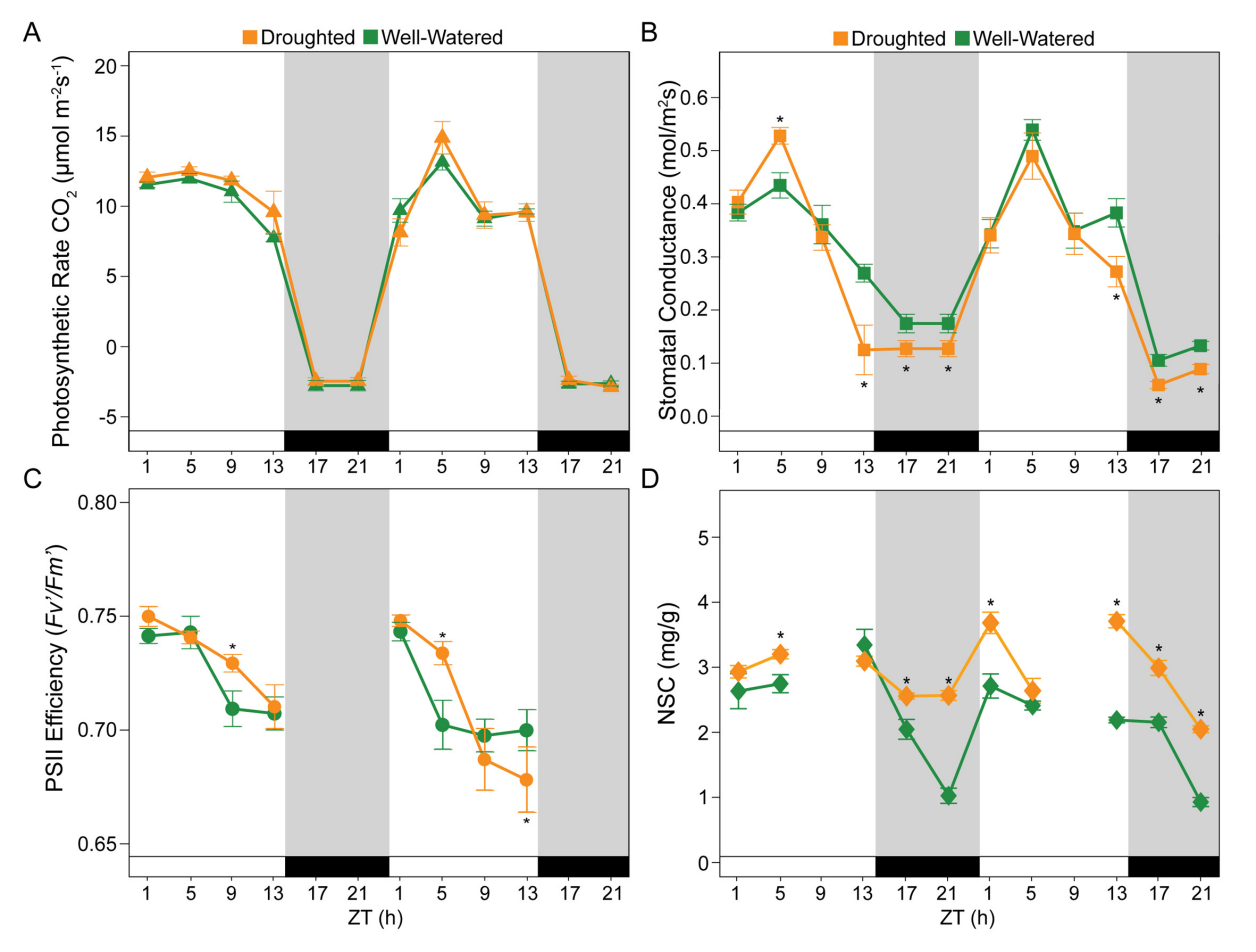
Specific time-of-day differences in physiology and in dynamics of Non-Structural Carbohydrates (NSC) were observed in *B. rapa* R500 plants subjected to mild drought relative to control plants. (**A**) Photosynthetic rate, *A* (μmol CO_2_ m^−2^s^−1^) of well-watered controls (green) and mild droughted (orange) plants. (**B**) Stomatal conductance, *g*_s_ (mmol H_2_O m^−2^s^−1^) of well-watered controls (green) and mild droughted (orange) plants. (**C**) Maximum efficiency of PSII in light adapted conditions, *Fv’/Fm’* of well-watered controls (green) and mild droughted (orange) plants. (**D**) NSC accumulation in well-watered (green) and mild droughted (orange) plants. NSC content is expressed as dry weight and refers to the sum of leaves and cotyledon biomass. Tissue collection was carried out every 4 hr during the 2 day time course except for ZT9 when no sugar extraction was performed. White and black bars and gray shading represent the dark period in the growth chambers (14 hr/10 hr; Day/Night). All represented values are averages of at least eight replicates ± SE, asterisks represent a significant difference (p<0.01) between treatments. See Figure 3—figure supplement 1. 10.7554/eLife.29655.008Figure 3—source data 1.Physiology measurements of well-watered (WW) and droughted (**D**) plants during Day 3 and Day 4 of the drought time course.

We observed diurnal changes in gas exchange in the well-watered plants, expressed as WUE and as its components *A* (Figure 3A, Figure 3—figure supplement 1) and *g*_s_ (Figure 3B, Figure 3—source data 1). As expected based on a previous analysis (Medrano et al., 2012), WUE did not provide an accurate measure of the plant response to drought stress due to limited drought severity and duration (Figure 3A, Figure 3—figure supplement 1). On both Day 3 and Day 4 of drought, *A* peaked at Zeitgeber Time (ZT) five where ZT0 corresponded to lights on (Figure 1A). At this time point, we recorded a net CO_2_ uptake of 12 ± 2 μmol m^−2^s^−1^ (Figure 3A). Droughted plants had photosynthetic capacity similar to well-watered plants for the duration of the experiment (Figure 3A), in agreement with previous studies of anisohydric plants where overall photosynthetic capacity was not disturbed by mild drought stress (Chaves et al., 2009; Cornic and Massacci, 1996; Flexas and Medrano, 2002). In contrast, there were significant differences in *g*_s_ between droughted versus well-watered plants with a reduction of 50% late in the day (ZT13) and 30% at night (between ZT17 and ZT21) on both Day 3 and Day 4 (Figure 3B). Thus, in late afternoon and at night *g*_s_ responds to small changes in soil water potential and seems to play an important role in the early response to drought. Our results show that *B. rapa*, like many crops, can reduce CO_2_ supply before *A* is impacted (Edwards et al., 2016).

**Figure 3—figure supplement 1.**
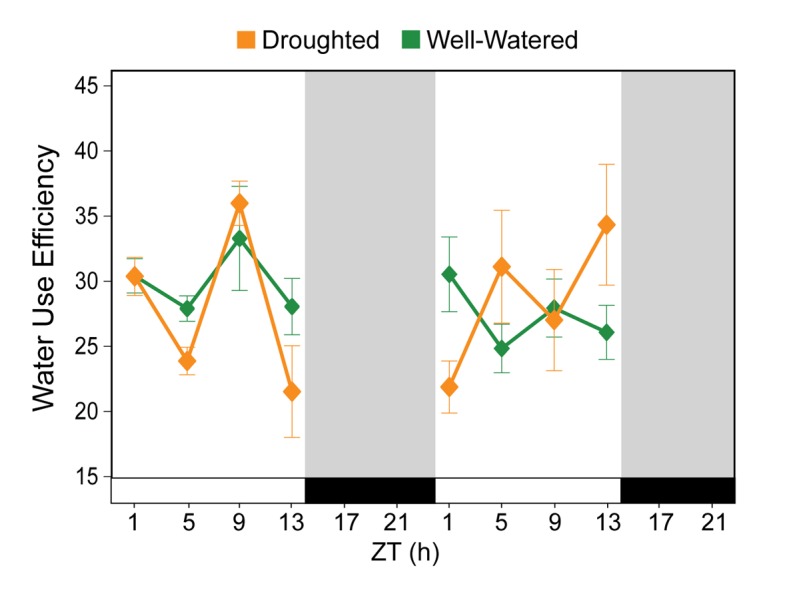
Water use efficiency (WUE) is not an accurate measure of plant response to drought. Variation of intrinsic WUE (as *A*/*g*_s_) for well-watered controls (green) and mildly droughted (orange) plants. Gray shading indicates dark period.

Our findings are consistent with studies showing that plants can lose as much as 30% of the daily water budget overnight (Dawson et al., 2007; Caird et al., 2007). Night transpiration is hypothesized to occur to enhance nutrient uptake (Matimati et al., 2014) and responds quickly to atmospheric and soil drought (Neumann et al., 2014; Schoppach et al., 2014) as shown here. It is likely that the low nighttime *g*_s_ observed in R500 plants (Figure 3B) contributed to the maintenance of turgor throughout the four days of drought (Figure 2B). Plants were still far from the wilting point (between −1.7 and −2 MPa) for R500 (Guadagno et al., 2017).

Although signaling mechanisms are not fully understood, diurnal patterns of *g*_s_ are sensitive to rapid changes in leaf water potential, causing both *g*_s_ and leaf hydraulic function to decline under stress (Brodribb and Cochard, 2009; Domec et al., 2009) with ABA synthesis as a major control over anisohydric responses (Brodribb and McAdam, 2013). Although an understanding of the relationships between the circadian clock, night transpiration, and nutrient uptake would dramatically improve predictive understanding of drought, information is scarce on how anisohydric plants behave at night in drought conditions (Rogiers et al., 2009; Klein, 2014; Martínez-Vilalta et al., 2014; Attia et al., 2015).

The maintenance of photosynthetic capacity in droughted plants despite the significant decrease in *g*_s_ may be partly explained by *Fv’/Fm’*, which was significantly greater for droughted than well-watered plants on both Days 3 and 4 of drought during the middle of the light period (Figure 3C). *Fv’/Fm’* presented a diurnal pattern with the highest values early in the day (ZT1 and ZT5) in both droughted and well-watered plants. Elevated *Fv’/Fm’* fully compensated for reduced gas exchange under mild drought conditions. Our results are consistent with recent work showing that the circadian clock optimizes photosynthetic capacity by modulating temporal dynamics of *Fv’/Fm’* (García-Plazaola et al., 2017).

### Dynamics of non-structural carbohydrates under drought

As expected, non-structural carbohydrates (NSC) accumulated during the day and decreased during the night in well-watered conditions (Figure 3D). In droughted plants, NSC levels were elevated throughout the night compared to well-watered controls. The presence of above-ground NSC accumulation suggested that the reduction in biomass observed (Figure 2A) was not due to a reduction in carbon availability but rather to the decreased nighttime conductance that preserves leaf turgor and high water potential (Chaves, 1991) at the cost of sugar translocation to growing tissues (Sevanto, 2014). That NSC levels were elevated at night in droughted plants highlights the close association between water use and carbon dynamics. Specifically, early perception of drought will influence carbon allocation by lowering gas exchange and respiration rate as we observe (Figure 3A). As previously reported, the lower level of respiration led to a decrease in biomass accumulation, and carbon remains in the chloroplasts because of slower transport of sugars out of the leaves (McDowell, 2011). Our results are supported by previous studies under fluctuating environmental conditions in which sugars such as glucose, fructose, and sucrose play a crucial role in maintaining cell turgor and vascular integrity in more extreme drought conditions than those studied here (Volaire, 1995; Sala et al., 2012).

Our time course analysis revealed physiological drought responses between ZT13 and ZT21 of each day, with higher magnitude on Day 4 than on Day 3. Early in drought, plants had lower *g*_s_ and higher levels of NSC in the above-ground tissues with respect to well-watered plants (Figure 3B,D). We found *g*_s_ to be the best physiological indicator of the early perception of drought stress in the plant, consistent with the view that *A* and *g*_s_ are regulated separately (Dodd et al., 2004; von Caemmerer et al., 2004) and that small decreases in *g*_s_ do not lead directly to reductions in *A* under mild drought.

### Co-expression network analysis reveals extensive temporal regulation of transcript level differences in well-watered and droughted plants

In parallel with the leaf physiological measurements, transcriptomic analysis (RNA-seq) was performed on leaf tissue to capture the temporal changes in transcript levels during the initial stages of drought. The breadth of circadian and diel regulation of gene expression results in time-of-day-dependent changes in the transcriptome (Harmer et al., 2000; Covington et al., 2008; Michael et al., 2008). Consequently, the response to abiotic stress, and in particular drought, has been shown to be dependent on the time of day in *Arabidopsis* and poplar, with the maximal transcriptome response occurring late in the day (Dubois et al., 2017; Wilkins et al., 2009; Wilkins et al., 2010) as was found in the physiological traits (Figure 3). To capture the diel transcriptome changes in the early stages of drought we applied a weighted gene co-expression network analysis (WGCNA, Langfelder and Horvath, 2008, Langfelder and Horvath, 2012Langfelder and Horvath, 2012) approach to classify genes based on their expression patterns throughout the day.

**Figure 4.**
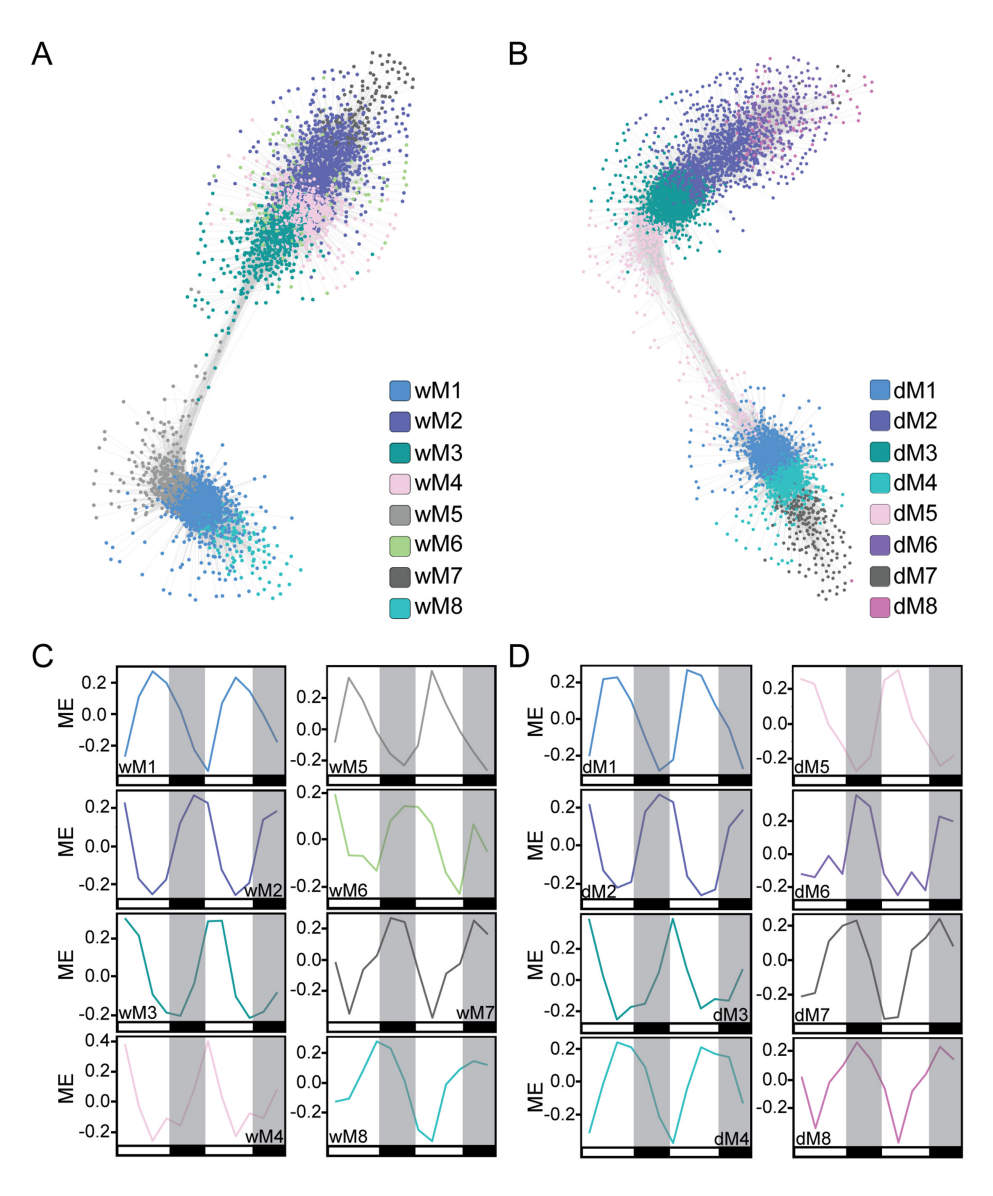
Co-expression network analysis identifies modules of temporally regulated genes with similar phase patterns. (**A and B**) The well-watered (**A**) and droughted (**B**) networks are shown with gene nodes colored by module membership. Network visualization was done in Cytoscape using a ForceDirected layout with an edge threshold cutoff of 0.1. Modules with significant overlap of gene membership between the well-watered and drought networks are similarly colored. (**C and D**) The module eigengene (ME) plots for the well-watered (**C**) and droughted (**D**) modules reveal temporal regulation of the top eight modules in each network containing ~80–85% of expressed genes. The ME plots are colored based on the modules in the network visualizations. White and black bars along the x-axis represent the day and night time points respectively.

**Figure 5.**
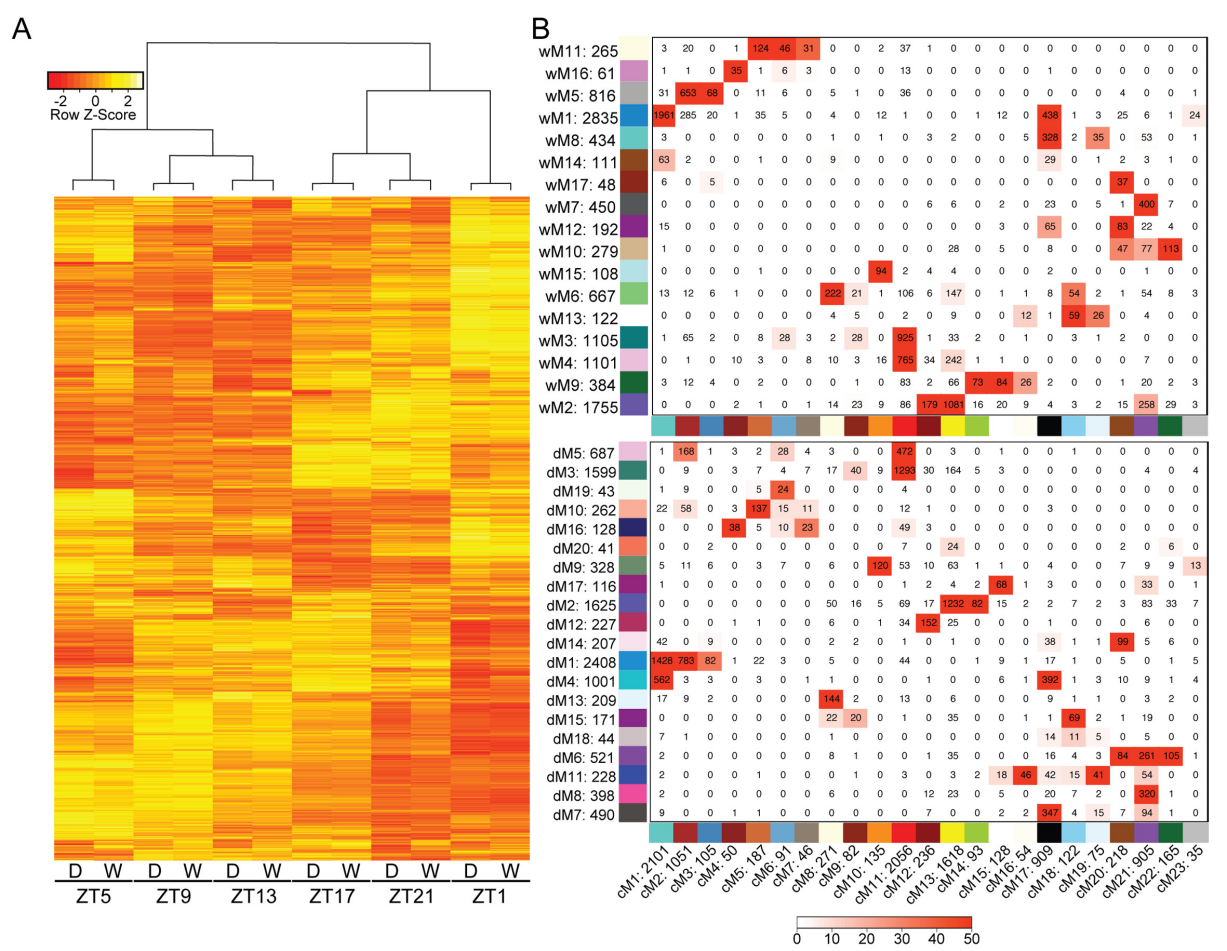
Variation in gene expression over a day is greater than the effects of mild drought. (**A**) Hierarchical clustering was performed on the log2 FPKM values for the Day 4 time points for well-watered and droughted plants. (**B**) Consensus network matrix for the well-watered (top) and droughted (bottom) networks. Consensus modules are listed along the bottom of both matrices with the well-watered and droughted modules along the side. Numbers beside the module number are the total gene counts for that module. Numbers inside the matrix are the number of genes in common between the two modules. Red numbers have significant overlap in gene count based on Fisher’s exact test with the –log(p) of the p-value encoding the coloring (0 = -log(1) and 50 = -log(1E-50)).

We first generated well-watered and droughted networks and examined the module eigengenes, or principal components, of the gene profiles for each module in the two networks. Not surprisingly, the top eight modules, containing 80–85% of the genes in the network analysis (see Materials and methods, Supplementary file 1), showed strong temporal expression patterns across the two-day time course (Figure 4). Previous studies have shown that time-of-day effects on the transcriptome are often greater than the effect of stress treatment (Wilkins et al., 2010). We performed hierarchical clustering of Day 4 samples from droughted and well-watered plants. In agreement with time-course transcriptome studies in *Arabidopsis*, poplar, and soybean (Wilkins et al., 2009; Wilkins et al., 2010; Rodrigues et al., 2015; Dubois et al., 2017) samples clustered based on time of day, rather than treatment, revealing that the transcriptome varied more with time of day than due to drought (Figure 5A). To examine the conservation in network topology between the droughted and well-watered transcriptomes, a consensus network to identify modules shared between the two networks was generated as previously described (Langfelder and Horvath, 2008). The consensus network contained significant overlaps in module classifications between the droughted and well-watered networks, consistent with the strong diurnal effects on the transcriptome (Figure 5B).

**Figure 6.**
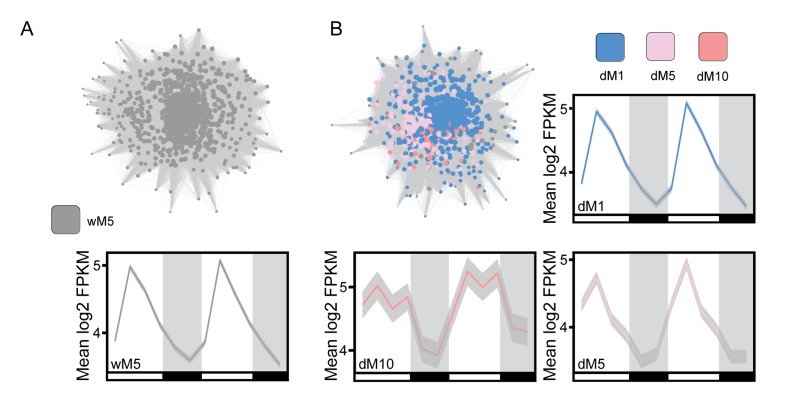
Expansion of gene expression patterns uncovered in the droughted network. (**A**) The wM5 module from the well-watered network is shown with the mean log2 FPKM expression profiles for the genes in the wM5 network that overlap with dM1, dM5, and dM10 modules. (**B**) The nodes in the wM5 module are highlighted based on their membership in the dM1, dM5, and dM10 modules from the droughted network. The mean log2 FPKM expression values for the genes in common between the wM5 module and each droughted module is shown. Grey shading in the expression profile plots represents the standard error of the mean log2 FPKM expression levels. White and black bars along the x-axis represent the day and night time points, respectively.

The well-watered and droughted networks contained 17 and 20 modules, respectively, suggesting that there are additional expression patterns in the droughted network due to rearrangement of the transcriptome in response to the drought treatment. For example, module 5 from the well-watered network (wM5) contained genes with expression patterns that produce a peak in transcript levels at ZT5 (Figure 6A). Roughly 95% of the genes in the wM5 module resolved into three distinct droughted modules, highlighted by the different color nodes in the network view (Figure 6B). The mean expression of the genes in the droughted modules revealed a change in expression pattern upon drought treatment (Figure 6B). The droughted module 5 (dM5) appeared to be most similar to the wM5 profile, whereas dM1 showed a shift in the phase of the time of lowest transcript level and dM10 shows a bi-phasic expression peak in both days (Figure 6B). Extensive rearrangement of the transcriptomic network, shown graphically in Figure 4, occurred as expected for anisohydric plants adjusting to a mild drought (Dal Santo et al., 2016). Similarly colored modules between the well-watered and droughted networks contained a significant overlap of genes with a common consensus network module (Figure 5B), consistent with their similar eigengene profiles (Figure 4C,D). As demonstrated by the network views (Figure 4A,B), there was visible rearrangement of the genes within the overlapping modules (wM2-4 compared to dM2-5). The co-expression network approach successfully incorporated time-of-day information to group genes based on their diurnal patterns of expression providing a more integrated view of the well-watered and drought transcriptomes.

### Correlating network modules with phenotypic traits

**Figure 7.**
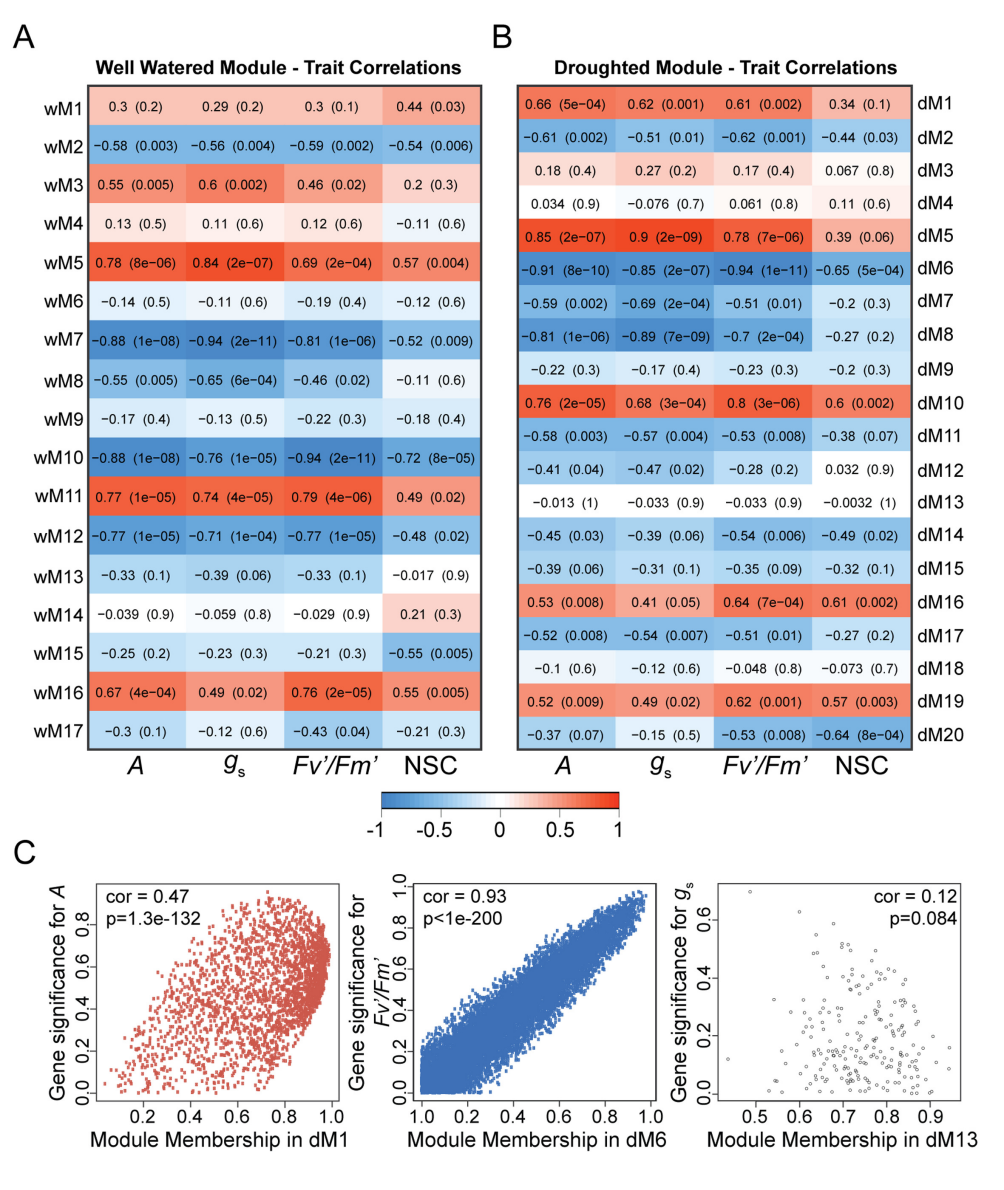
Dynamic changes in physiology are correlated with co-expression networks. (**A and B**) Correlations between module eigengenes and physiology measurements for well-watered (**A**) and droughted (**B**) plants. The numbers within the heat map represent correlations and *P* values (in parentheses; red, positively correlated, blue, negatively correlated) for the module – trait associations. (**C**) Scatterplots of gene significance versus module membership for the photosynthetic rate with dM1 (red), PSII efficiency with dM6 (blue), and stomatal conductance with dM13 (black). Significant correlations imply that hub genes within the module are also highly correlated with the physiology measure.

To relate the gene expression modules to the physiology time-course data, we used WGCNA to correlate the module eigengenes with the mean values of each individual physiology measurement (*A, g*_s_, NSC, and *Fv’/Fm’)* at each time point. Gene significance measures were calculated as the absolute value of the correlation with the physiological data (Supplementary file 1, Horvath et al., 2006; Fuller et al., 2007). Several modules in both networks were positively or negatively correlated with various physiological measurements (Figure 7A,B). Modules in both networks with similar phasing had similar trait correlations. For example, the wM5 and dM5 modules with peak expression between ZT1-5 were positively correlated with the *A, g*_s_, and *Fv’/Fm’*, which had similarly phased peaks (Figure 7A,B). Conversely, wM7 and dM8, which peak around ZT17, were negatively correlated with *A, g*_s_, and *Fv’/Fm’*. Both sets of modules had a significant overlap of genes with consensus module 21 (Figure 5B). wM11, wM16, and dM10 were positively correlated with and wM7, wM10, wM12, dM6, and dM8 were negatively correlated with *A, g*_s_, NSC, and *Fv’/Fm’* (Figure 7A,B). Within the modules there were genes that had high gene significance measures with the physiology and high module membership with the module eigengenes (Figure 7C). The similar correlations observed for both well-watered and droughted networks are to be expected with a treatment that causes mild changes to physiology; however, we did observe significant differences in *Fv’/Fm’*, *g*_s_, and NSC measurements in response to drought, suggesting that these traits are valid predictors of the early perception of drought stress in the plant when sampled throughout the day. We focused on these traits and selected the modules in the droughted network that were significantly correlated with these measures.

### Applying a circadian-guided approach to identify drought responsive genes

Many of the genes within the modules in the droughted network that were significantly correlated with the physiology data were also correlated in the well-watered network making it difficult to identify drought-specific changes. The rhythmic patterns of gene expression and physiology inherent in the data make it amenable to circadian data analyses. In order to identify genes that are differentially expressed in response to drought we applied a circadian transcript analysis program, JTK-CYCLE, to compare the rhythmic profiles of the genes within the modules of interest between the two networks. JTK-CYCLE is a non-parametric statistical algorithm designed to identify circadian regulated transcripts and estimates period, phase, and amplitude (Hughes et al., 2010). The genes within the droughted network modules that were positively (dM1, dM5, dM10, dM16, and dM19) or negatively (dM2, dM6, dM7, dM8, dM11, dM14, dM17, and dM20) correlated with *g*_s_ and *Fv’/Fm’* (Figure 7B) with p<0.01 (Supplementary file 1) were selected for analysis. The expression levels from both the well-watered and droughted datasets were used for JTK-CYCLE with period parameters set at 24 hr since our data was collected under 24 hr light/dark cycles. Genes were classified as rhythmic using a cut-off q-value <0.01 (Supplementary file 2).

**Figure 8.**
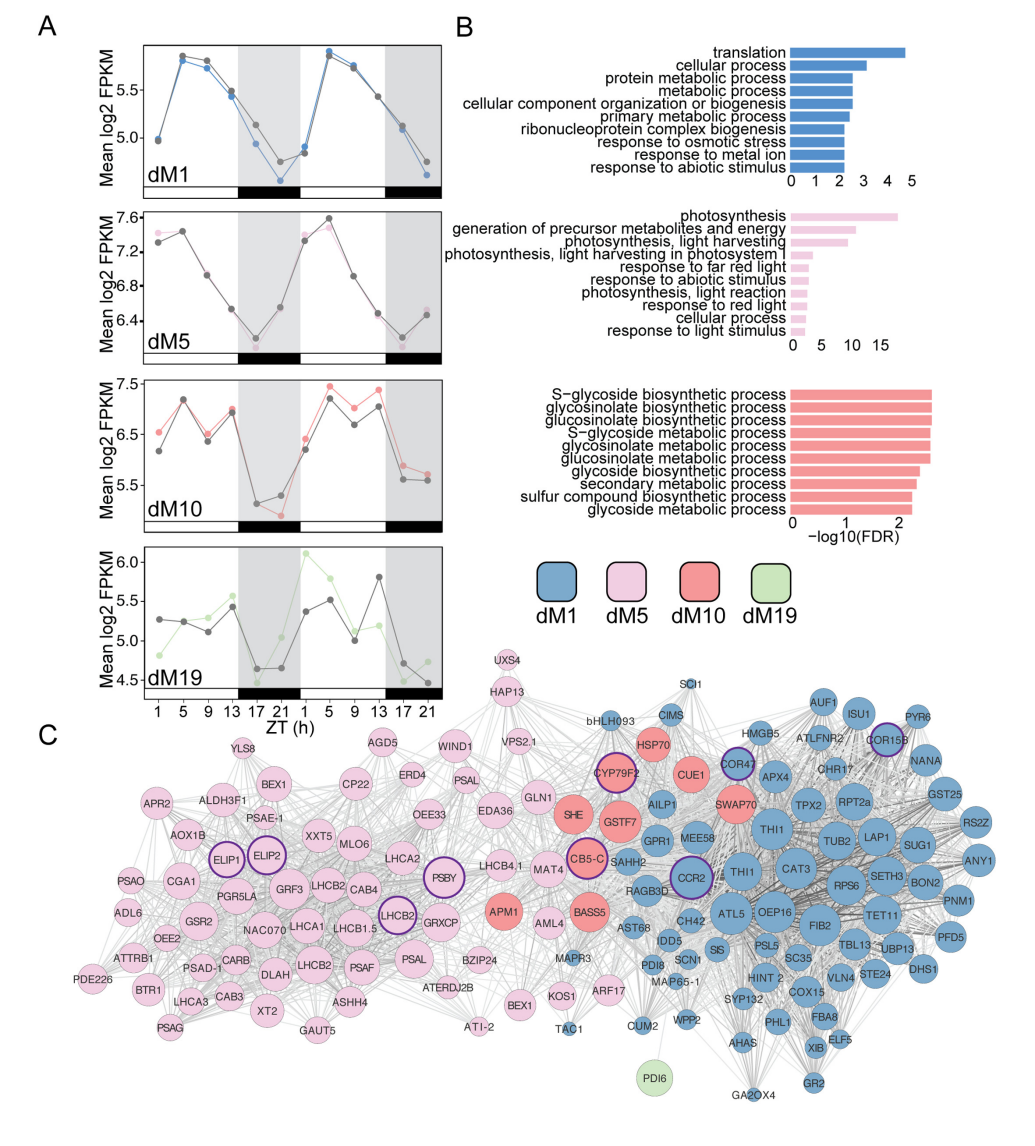
Modules positively correlated with physiology are associated with metabolism and light harvesting processes. (**A**) Mean log2 FPKM expression profiles of genes in the dM1, dM5, dM10, and dM19 modules that are positively correlated with stomatal conductance and identified by JTK-CYCLE as having an amplitude change between well-watered and droughted samples. Grey lines are always well-watered. White and black bars along the x-axis represent the day and night time points, respectively. (**B**) Top 10 associated GO terms for dM1 (top), dM5 (middle), and dM10 (bottom). (**C**) Genes identified in the dM1, dM5, dM10, and dM19 modules with known *Arabidopsis* orthologs are shown in the network view. Nodes are colored based on module and size based on module membership. The larger nodes are highly connected within a module and have greater ‘hubness’. The nodes circled in purple were validated by Nanostring.

Drought-responsive candidate genes were identified based on two criteria. First, we selected transcripts that were not rhythmic in the well-watered dataset but which became rhythmic upon the imposition of drought. Second, among the transcripts that were rhythmic under both conditions, we were interested in transcripts that changed (either increased or decreased) in amplitude of expression upon the imposition of drought. To identify these transcripts we calculated the difference in amplitude for each transcript between the droughted and well-watered datasets and chose transcripts with an amplitude difference greater than 10 for further analysis (Supplementary file 2). To examine the expression change for the selected genes we re-grouped them based on their modules in the droughted network and plotted the mean expression profiles of these genes for each module. We first examined the positively correlated modules (Figure 8A). The log2 mean expression profiles of dM1 and dM5 genes exhibit peak expression levels at ZT5 as do the *g*_s_ data, consistent with the positive correlation of these modules with *g*_s_. In both modules, genes appeared to be down regulated at the end of the light period and into the night for dM1 and down regulated early in the night for dM5. The dM10 module, which was correlated with *Fv’/Fm’*, showed an elevated level of expression on Day 4 relative to Day 3, consistent with the elevated *Fv’/Fm’* in droughted plants on Day 4 (Figure 3C).

To validate the biological relevance of the selected genes from these modules, we analyzed the top 10 enriched GO categories for the positively and negatively correlated module lists containing at least five genes with *Arabidopsis* syntenic orthologs. The dM1 module was enriched for primary metabolism and response to abiotic stimulus (Figure 8B,C). dM5 was enriched for photosynthesis, response to light, and abiotic stress stimulus (Figure 8B,C). Abiotic stress response is expected under mild drought in anisohydric plants because the mesophyll cells are exposed to lower water potentials earlier in the drought than in isohydric plants (Dal Santo et al., 2016). Interestingly, the dM10 module with the bi-phasic peaks contained genes involved in glucosinolate biosynthesis and metabolism (Figure 8B,C). Previous work has shown that abiotic stress leads to an increase in secondary metabolism that is likely the result of carbon reallocation (Del Carmen Martínez-Ballesta et al., 2013). At Day 4, the stage in the mild drought treatment at which *Fv’/Fm’* was beginning to decrease, the transcript data suggested that the plant is altering glucosinolate production. Although the exact purpose of this response is unclear (Del Carmen Martínez-Ballesta et al., 2013), growth-defense tradeoffs are expected when stress reduces growth (Züst and Agrawal, 2017) and secondary metabolism alterations can change circadian clock outputs (Kerwin et al., 2011) that potentially include drought responses.

**Figure 9.**
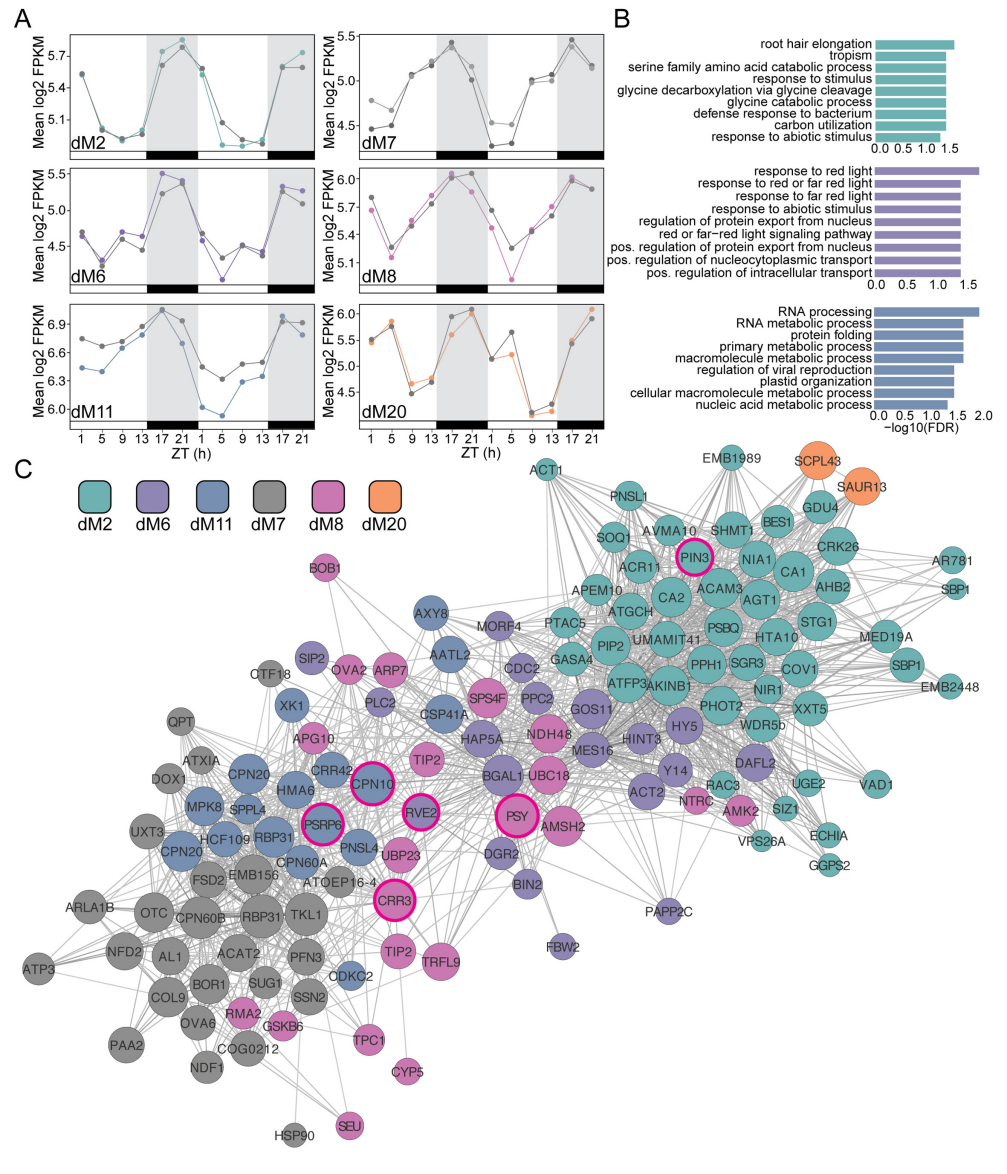
Modules negatively correlated with physiology are associated with abiotic stress and light response. (**A**) Mean log2 FPKM expression profiles and of genes in the dM2, dM6, dM7, dM8, dM11, and dM20 modules that are negatively correlated with stomatal conductance and identified by JTK-CYCLE as having an amplitude change between well-watered and droughted samples. Grey lines are always well-watered. White and black bars along the x-axis represent the day and night time points, respectively. (**B**) Top 10 associated GO terms for dM2 (top), dM6 (middle), and dM11 (bottom). (**C**) Genes identified in the dM2, dM6, dM7, dM8, dM11, and dM20 modules with known *Arabidopsis* orthologs are shown in the network view. Nodes are colored based on module and size based on module membership. The larger nodes are highly connected within a module and have greater ‘hubness’. The nodes circled in magenta were validated by Nanostring.

We next examined the modules that were negatively correlated with *g*_s_ and *Fv’/Fm’*. Consistent with the significant decrease in *g*_s_ on Day 4 in droughted plants compared to well-watered plants, the genes in these modules showed a decrease in expression on Day 4 and in the case of dM6 an increase in expression on Day 3 as well (Figure 9A). Interestingly, dM6 and dM8 displayed slight phase shifts in expression pattern with an earlier peak in expression on Day 3 compared to Day 4 suggesting that these genes contribute to the initial stages of the drought response. The genes in these modules are related to photosystem efficiency and light response pathways (Figure 9B,C), consistent with the decrease in *Fv’/Fm’* observed on Day 4. dM7, dM11, and dM17 show dramatic decreases in expression on Day 4 relative to well-watered plants and contain genes involved in nitrogen metabolism, amino acid biosynthesis, and phosphatase activity (Figure 9B,C).

Comparing circadian features proved to be an effective way of identifying genes with altered patterns in the droughted relative to the well-watered network as seen by the significant GO enrichment of the selected genes (Figures 8 and 9) that not only validates the biological relevance of the module structure but also the potential importance of the selected genes within these pathways. For this analysis, we chose an amplitude change of 10 as a cutoff based on an initial screen of rhythmic gene expression profiles but there are likely to be genes outside of this cutoff that exhibit a biologically meaningful change and genes within the list that do not. To validate some of the identified genes, we compared the expression levels in five biological replicate plants for each treatment, harvested during the drought experiment, without pooling of tissue from multiple plants as was done with the RNA-seq experiment. One of the limitations of time-course experiments is the cost associated with sequencing each time point at high replication. Using the JTK-CYCLE filtered gene list, we ranked the genes based on their module membership and selected the top three genes from the modules correlated with the physiology data (Figures 8 and 9). In addition, we selected genes from the list with GO ontologies associated with abiotic stress response and light harvesting processes. We identified a list of 36 genes for validation using the NanoString PlexSet technology.

**Figure 10.**
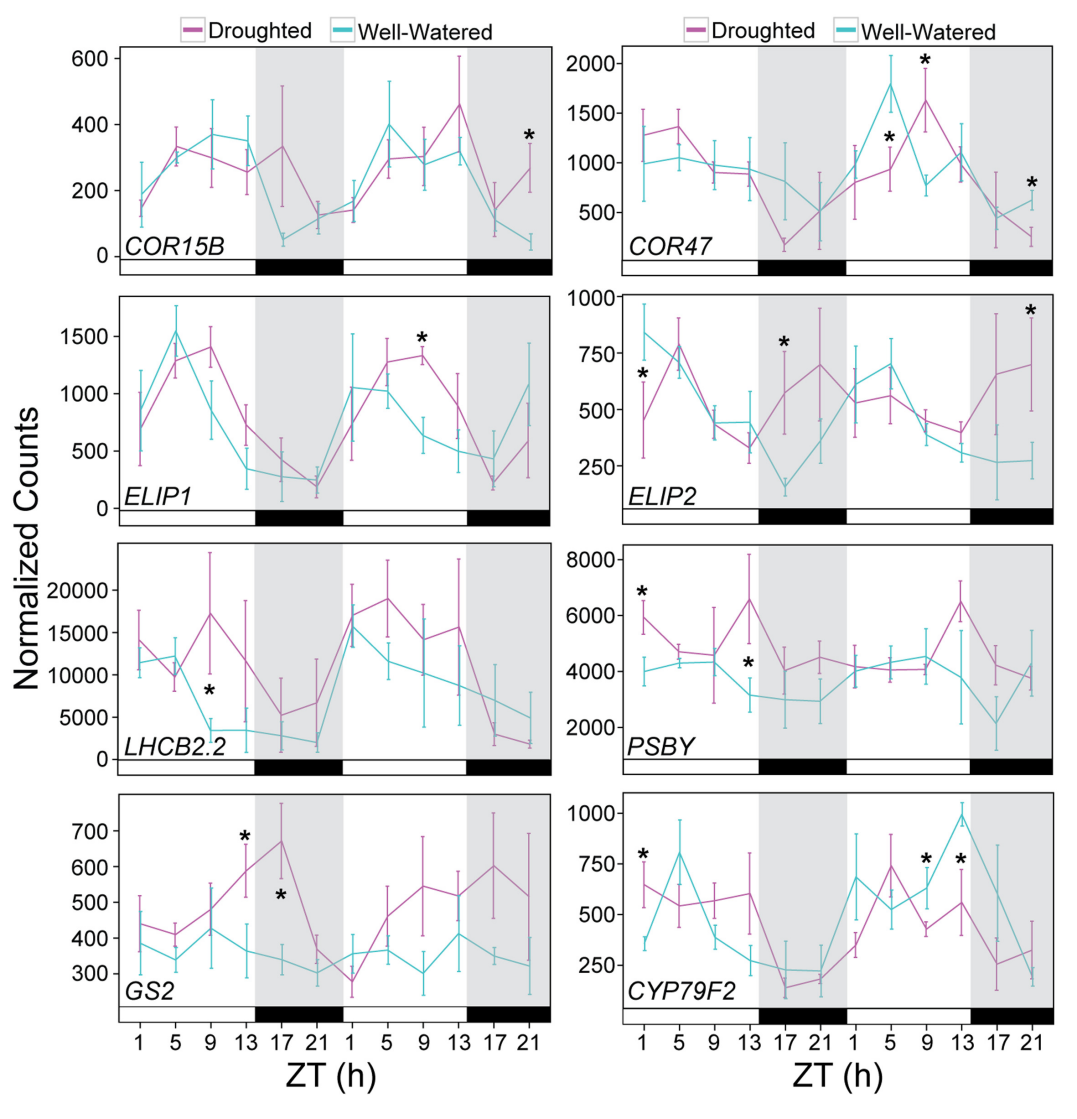
Validation of drought-responsive genes supports alterations of light harvesting processes and metabolism. Gene expression for a set of drought-responsive genes identified from the JTK-CYCLE filter. NanoString was performed on RNA isolated from leaf tissue harvested during the RNA-seq time course experiment. Each data point represents the mean of 5 individual plants. Gene counts were normalized to *Bra021441, Bra014841*, and *Bra020305.* Error bars represent SE. Grey shading indicates the dark period. Asterisks indicate significant difference (p<0.05). See Figure 10—figure supplement 1. 10.7554/eLife.29655.018Figure 10—source data 1.List of genes selected for gene expression validation using NanoString.

The NanoString data supported the trends observed in the RNA-seq dataset. The diel expression patterns seen in the well-watered and droughted plants and specific time-of-day responses to drought were recapitulated for the genes evaluated (Figure 10, Figure 10—figure supplements 1 and 2, Figure 10—source data 1). The expression of two members of the C-repeat-binding factor (CBF) regulon *COR15B* and *COR47* (dM1, Figure 8C) showed increased and shifted peak expression on Day 4 of droughted plants relative to well-watered plants (Figure 10) consistent with their known roles in abiotic stress response (Novillo et al., 2007). Consistent with the increase in *Fv’/Fm’*, several genes related to light harvesting and photosystem regulation showed elevated expression levels during the day in droughted plants. The *EARLY LIGHT-INDUCIBLE PROTEIN 1* and *2* (*ELIP1/2*) genes (Figure 8C), members of chlorophyll *a/b* – binding (CAB) protein superfamily and postulated to be photoprotectants for PSII under various stress conditions (Hayami et al., 2015) were both elevated in expression level and showed phase-delayed expression profiles (Figure 10). Components of PSII, *LIGHT-HARVESTING CHLOROPHYLL B-BINDING 2* (*LHCB2.2*) and *PHOTOSYSTEM II BY* (*PSBY*), also exhibited elevated expression during the day (Figure 8C, Figure 10). As with all studies that use a correlation in time to study gene expression to trait relationships, we could not address gene expression to trait relationships that take longer than 4 hr, our sampling frequency. Similarly, we note that changes in transcript abundance do not inevitably result in changes in protein abundance or activity and will not identify meaningful changes resulting from post-translational regulation (Graf et al., 2017).

**Figure 10—figure supplement 1.**
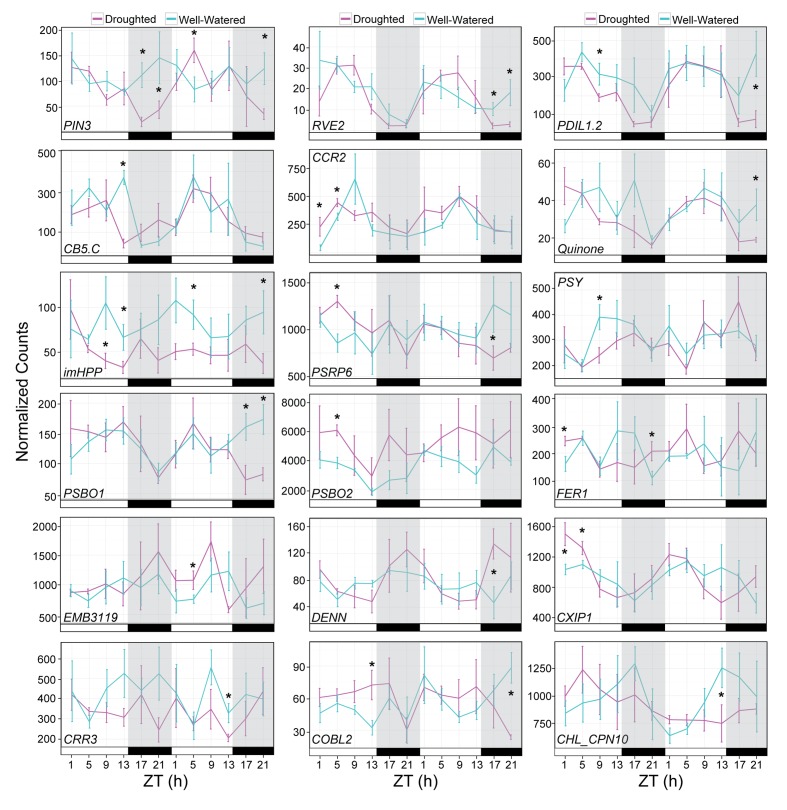
Validation of drought responsive genes. Gene expression for a set of drought-responsive genes identified from the JTK-CYCLE filter. NanoString was performed on RNA isolated from leaf tissue harvested during the RNA-seq time course experiment. Each data point represents the mean of 5 individual plants. Gene counts were normalized to *Bra021441, Bra014841*, and *Bra020305.* Error bars represent SE. Black bars and grey shading indicates the dark period. Asterisks indicate significant difference (p<0.05).

**Figure 10—figure supplement 2.**
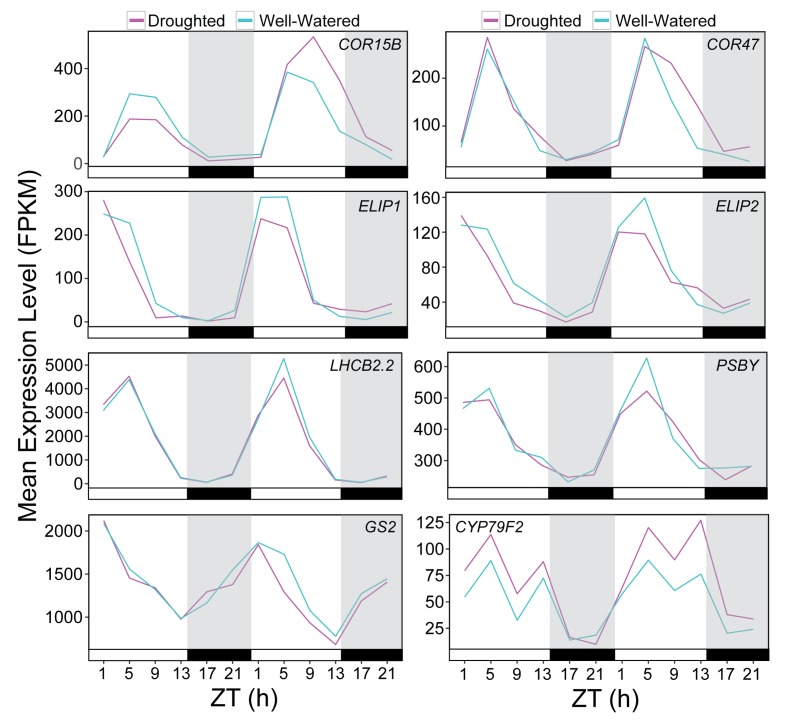
RNA-seq expression of drought responsive genes assessed by NanoString. FPKM expression values for the drought responsive genes analyzed by Nanostring and shown in Figure 10. Each data point represents the mean of two biological replicates. Black bars and grey shading indicates the dark period.

In addition to changes associated with light responses, altered expression for genes involved in nitrogen metabolism was confirmed. The *GLUTAMINE SYNTHETASE 2* (GS2) gene, encoding the light- and CO_2_-induced chloroplastic glutamine synthetase GS2 that assimilates ammonium produced during photorespiration and nitrite reduction (Taira et al., 2004) was elevated late in the day in droughted plants (Figure 10, Figure 10—figure supplement 2). An overall reduction in expression of the mRNA encoding an integral membrane HPP family protein predicted to transport nitrite into plastids (Maeda et al., 2014) was observed in droughted plants (Figure 10—figure supplement 1). The decrease in nitrate transport is consistent with a decrease in nutrient uptake, and the increase in *GS2* levels may be a response to ammonium produced from an increase in photorespiration. The drop in *g_s_* during the night and the accumulation of NSC in droughted plants on Day 4 of the treatment coincided with decreased expression of the gene encoding CYP79F2, which metabolizes long-chain aliphatic glucosinolates (Figure 8C, Figure 10). The nitrogen metabolism changes are consistent with the role of night transpiration in nitrogen uptake (Matimati et al., 2014) and the constitutive nature of nitrogen uptake and assimilation compared to other nutrients (Hole et al., 1990) and suggest a fruitful line of research on interactions among drought, nitrogen uptake and assimilation, and the circadian clock. Knockdown of *CYP79F2* using RNAi in *Arabidopsis* led to a drop in aliphatic glucosinolates and an increase in indole glucosinolates as well as storage carbohydrates such as fructose and galactose in addition to changes in several hormone levels (Chen et al., 2012). The significant drops that we observed in *CYP79F2* expression occurred at the ZT9 and ZT13 time points on Day 4 of the treatment when sugar accumulation was observed (Figures 3D and 10). These temporal changes in gene expression are examples of the rearrangements seen in the drought network and offer new insights into the dynamic transcriptome level changes occurring following early drought perception in *B. rapa*.

In this study we characterized the onset of drought response by using temporal changes in physiology to support the biological significance of transcriptome changes. This approach validated the need for time-of-day resolution to observe the dynamic changes in physiology and to filter out the diel changes that cause transcript abundance variations independent of treatment. Integrating these dynamic changes in physiology with the transcriptome data using a circadian-guided network approach uncovered changes in expression of several photosynthetic and metabolic genes, suggesting an early sensing of the drought treatment at the molecular level. Future work is needed to compare the time-of-day dependent drought response of these genes in genetically and phenotypically diverse plants in order to associate the unique transcript dynamics with specific physiological responses to drought.

## Materials and methods

### Plant material and growth conditions

Seeds of *Brassica rapa* subsp. *trilocularis* (Yellow Sarson) R500 were planted in pots (156 cm^3^) filled with a soil mix (Miracle-Gro Moisture control Potting Mix (20% v/v), Marysville, OH, and Profile Porous Ceramic (PPC) Greens Grade (80% v/v), Buffalo Grove, IL) amended with 2 ml of Osmocote 18-6-12 fertilizer (Scotts, Marysville, OH) per pot. Plants were randomized per treatment into four growth chamber compartments (PGC-9/2 Percival Scientific, Perry, IA). Chambers were set to a 14 hr/10 hr (day/night) photoperiod with a photosynthetic photon flux density (PPFD) at the plant height of ~130 μmol photons m^−2^ s^−1^. Temperature was set to 21°C (±2)/18°C (day/night) cycle with relative humidity maintained between 28–33%.

### Experimental design

Plants were watered daily to maintain moist soil conditions until 16 days after sowing (DAS) when water was withheld from half the plants (Droughted; Figure 1A). Sampling began two days after drought onset (18 DAS), with samples collected every 4 hr over 48 hr beginning 1 hr after lights on (ZT1) on the third day of drought (Figure 1A). The well-watered soils maintained soil water potential (*Ψ*_s_) between 0 and −0.5 MPa throughout the experiment (Figure 1B). *Ψ*_s_ declined progressively to −1.5 MPa over the 48 hr for the droughted plants (Figure 1B). Physiological data and leaf tissue for RNA-seq were collected in separate experiments performed under identical conditions (Figure 1B) in order to minimize duration of sampling and to avoid potential alterations of gene expression in response to perturbations associated with the physiological measurements. To assess whether the two experiments elicited similar physiological responses to drought, *Fv’/Fm’* was measured at 4 hr intervals during the day and above-ground biomass was determined at ZT17 on Day 3 and Day 4 for each experiment; neither showed any significant difference between the two experiments (Supplementary file 1). Accordingly, for these two traits, we pooled data from the replicate experiments (Figure 3—source data 1).

### Gas exchange

Photosynthetic rate (*A*) and stomatal conductance (*g*_s_) were measured on the youngest fully expanded leaves according to the protocol described by Long and Bernacchi (Long and Bernacchi, 2003) using three portable gas exchange systems provided with a 2 cm^2^ leaf chamber fluorimeter (LI-COR-6400XT; LI- COR Biosciences Inc., Lincoln, NE, USA). All spot measurements were taken in the same growth chamber compartment where plants were growing, and environmental conditions in the cuvette matched those in the growth chamber. The following conditions were set for the LiCOR measurements: flow rate, 300 μmol s^−1^; CO_2_ concentration, 400 μmol mol^−1^; VPD, 1.3–1.9 kPa; PPFD, 150 μmol photons m^−2^ s^−1^; leaf temperature, 22°C; and the cuvette fan was set to fast. Measurements in the dark (ZT14 through ZT24 on Day 3 and Day 4) were taken with the same cuvette settings except that a dim green light (~1 μmol photons m^−2^ s^−1^) was used. For each replicate, gas exchange values were recorded after stabilization of the readings (max 4 min). The intrinsic WUE was calculated as *A*/*g*_s_ according to Seibt *et al*. (Seibt et al., 2008).

### Chlorophyll *a* fluorescence

Chlorophyll *a* fluorescence (Humplík et al., 2015) was measured using a hand-held fluorimeter (Fluopen FP100, PSI, Brno, Czech Republic) as *Fv’/Fm’*, maximum efficiency of PSII in light conditions. The actinic light source of the FluorPen was maintained at ~200 μmol photons m^−2^ s^−1^. *Fv’/Fm’* was measured using a saturation pulse (0.800 s;~2200 photons μmol m^−2^ s^−1^). Calculations of *Fo'* used the following equation from Oxborough and Baker (Oxborough and Baker, 1997) where *Fo’=Fo/(FvFm +Fo/Fm')*. For the nighttime samples (ZT14 through ZT24 on Day 3 and Day 4), *Fv/Fm*, maximum efficiency of PSII in dark-adapted conditions, was measured as described previously (Murchie and Lawson, 2013); the measuring light of the FluorPen was set at ~1,500 μmol photons m^−2^ s^−1^ with a saturation pulse at ~2200 photons μmol m^−2^ s^−1^. All dark measurements were taken using a dim green light (~1 μmol photons m^−2^ s^−1^).

### Above-ground biomass

At ZT17 on Day 3 and Day 4, six replicate plants from each treatment were harvested for fresh and dry biomass measures. Above-ground tissue was cut at the soil level with a razor blade, weighed, oven-dried for 10 days at 65°C and weighed again for dry biomass.

### Non-structural carbohydrates

NSC were measured using the anthrone method (Seifter et al., 1950). Above-ground plant tissue (leaves and cotyledons) was collected, flash-frozen, and ground. The powder (~0.1 g), after air-drying, was extracted in 10 ml of 80% ethanol, incubated at 80°C for 30 min, and centrifuged for 5 min. The pellets were extracted two more times with 80% ethanol. An aliquot of the extract was hydrolyzed in 5 ml anthrone solution (4 g anthrone in 1000 ml 95% H_2_SO_4_; Sciencelab.com, Houston, TX) in a boiling water bath for 15 min. After cooling, the sugar concentration was determined spectrophotometrically at 620 nm using glucose as a standard.

### Statistical analysis for physiology data

We averaged all replicate samples for each physiological trait and calculated standard errors for each time point. The two treatments were compared at every time point using a one-tailed unpaired Student’s t-test.

### Collection of leaf tissue for RNA-sequencing

For RNA-seq, ~1 cm^2^ sections were cut from the youngest fully developed leaf and immediately flash frozen in liquid nitrogen. Preserved tissue was placed in long-term storage at −80°C until RNA extraction. At each time point, tissue from 10 plants in the same treatment was collected and five plants were pooled for each biological replicate, resulting in two biological replicates per treatment at each time point.

### RNA-sequencing library preparation and processing

We used a modified mRNA isolation protocol (Supplementary file 3) to isolate mRNA directly from *B. rapa* R500 leaf tissue. The mRNA was used to make strand specific libraries according to the low-cost library protocol from Wang *et al.* (Wang et al., 2011a). Library quality and size were verified using a 2100-bioanalyzer (Agilent Technologies, Santa Clara, CA). Libraries were pooled into 12 sample sets and sequenced across 4 lanes (12 time points/time course +2 replicates of each treatment = 48 libraries) as 101 bp paired-end reads using Illumina HiSeq2500 (Illumina, San Diego, CA). Raw data has been submitted to GEO (http://www.ncbi.nlm.nih.gov/geo) under accession number GSE90841. The raw fasta reads were filtered using trimmomatic (RRID:SCR_011848; http://www.usadellab.org/cms/index.php?page=trimmomatic) with mostly default settings (ILLUMINACLIP:./TruSeq3-PE-2.fa:2:30:10 LEADING:3 TRAILING:3 SLIDINGWINDOW:4:25 HEADCROP:14 MINLEN:50). Prior to aligning to the Chiifu reference genome (Wang et al., 2011b), existing R500 DNA-seq data (https://www.ncbi.nlm.nih.gov/sra; SRR065676) were used to call SNPs in the Chiifu genome using GATK (RRID:SCR_001876; https://www.broadinstitute.org/gatk/) with default settings. The vcf file generated by GATK was filtered to remove any SNPs with a quality score below 30, coverage below 10 and all heterozygous SNPs. The remaining SNPs were used to replace the Chiifu reference genome using the vcf-consensus tool in the VCFtools package (RRID:SCR_001235; http://vcftools.sourceforge.net/perl_module.html). Tophat2 (RRID:SCR_013035) was used to align the RNA-seq reads to the modified Chiifu genome file using default settings and first-strand library type. Transcripts were assembled using cufflinks with the Chiifu reference Brassica_rapa.IVFCAASv1.19.gtf annotation file. FPKM values were generated using cuffdiff (RRID:SCR_001647) with –-no-diff –-no-js-tests options (Trapnell et al., 2010; 2012).

### WGCNA network analysis

The well-watered and drought time course datasets were filtered to remove any genes that did not reach an FPKM value of 10 in at least one time point in order to remove non-varying or low-abundance genes that introduce noise into the network analysis. Log2 normalized FPKM values were used to generate the co-expression networks using the WGCNA (RRID:SCR_003302) package in R (Team RC, 2016; Langfelder and Horvath, 2008; Langfelder and Horvath, 2012). Independent signed networks were constructed from the well-watered and drought time-course samples. An adjacency matrix was constructed using a soft threshold power of 16. Network interconnectedness was measured by calculating the topological overlap using the TOMdist function with a signed TOMType. Average hierarchical clustering using the hclust function was performed to group the genes based on the topological overlap dissimilarity measure (1-TOM) of their connection strengths. Network modules were identified using a dynamic tree cut algorithm with minimum cluster size of 30 and merging threshold function at 0.25. To visualize the expression profiles of the modules, the eigengene (first principal component) for each module was plotted using ggplot2 in R. To identify hub genes within the modules, the module membership (MM) for each gene was calculated based on the Pearson correlation between the expression level and the module eigengene. Genes within the module with the highest MM are highly connected within that module. To relate the physiology measurements with the network, the module eigengenes were correlated with the physiology data. Correlations were performed for each physiology trait separately using the mean values at each time point to associate the diel patterns between the physiology and eigengenes. To associate individual genes with the physiology we calculated Gene Significance (GS) as described in the WGCNA package as the absolute value of the correlation between gene expression and physiology across the time series.

### NanoString sample preparation and analysis

To validate a subset of genes identified in the WGCNA and JTK-CYCLE analysis, five individual plants from the well-watered and drought conditions were collected during the time course experiment alongside the plants harvested for RNA-seq. Leaf tissue was ground in Lysis Binding Buffer (LBB) as described in the mRNA extraction protocol (Supplementary file 3). Following the centrifugation in LBB, 400 μl of lysate was used for RNA extraction using the Zymo Research Plant RNA MiniPrep kit (Zymo, Irvine, CA). RNA purity was assessed with a NanoDrop spectrophotometer (Thermo Fisher Scientific, Waltham MA), and concentration was determined using the Qubit broad range RNA assay kit according to the manufacturer’s instructions (Thermo Fisher Scientific, Waltham MA). An initial RNA titration test was performed for each probeset with 50 ng, 100 ng, 150 ng and 200 ng probe to optimize the concentration. We chose 150 ng for the full time course assay. All five replicate samples for each time point and treatment were randomly arranged across 96-well plates with a random set of technical replicates. The NanoString PlexSet assay was performed according to the manufacturer’s instructions (NanoString Technologies, Seattle, WA) at the Molecular Biology Core Facility at Dartmouth College. Normalization was performed using the NanoString nSolver Analysis Software 3.0 with default settings. The housekeeping genes selected for Content Normalization were *Bra021411*, *Bra014841*, and *Bra020305*. These genes were selected based on criteria including absence from the rhythmic modules and JTK-CYCLE list of cycling genes and low level of overall change in FPKM across the 2 day time course in both the well-watered and droughted samples. These genes also represent low, medium, and high expression levels. For the CodeSet normalization, a row of the plate containing technical replicates of two pooled RNA samples (droughted samples Day 3 ZT5 and Day4 ZT39) were used. Normalized data were exported and further analyzed in R. Based on the technical replicate comparisons it was evident that there are occasional spurious probe counts for a single gene within a sample that were not reproduced in the technical replicate indicating a technical problem rather than biological. To remove these probe counts we calculated the modified Z-score for each probe across all samples and removed probes above 3. For all samples with technical replicates we selected the sample with the lowest maximum modified Z-score. The five biological replicate samples were averaged, and standard errors calculated for each time point and a one-tailed unpaired Student’s t-test was performed to compared data from the well-watered and droughted samples at every time point.

## Additional files

Supplementary file 1. WGCNA network gene lists with module membership and gene significance for each physiology measure.

Supplementary file 2. JTK-Cycle analysis results for genes from trait correlated modules with gene significance p.value scores less than 0.01.

Supplementary file 3. mRNA Purification Protocol for RNA‐seq Library Preparation

Transparent reporting form

### Major datasets

The following dataset was generated:

Greenham KGuadagno CRGehan MAMockler TCWeinig CEwers BERobertson McClung C2016Network analysis identifies temporal regulation of transcriptomic and physiological responses to early drought perception in Brassica rapa.https://www.ncbi.nlm.nih.gov/geo/query/acc.cgi?acc=GSE90841Publicly available at the NCBIGene Expression Omnibus (accession no: GSE90841)
