# Supplementary material for: Temporal network analysis identifies early physiological and transcriptomic indicators of mild drought in Brassica rapa
Source: eLife. 2017 Aug 18;6:e29655. doi: 10.7554/eLife.29655 (PMC5628015; doi:10.7554/eLife.29655)
Supplement: Supplementary file 3. [file elife-29655-supp3.pdf]

1- mRNA Purification Protocol for RNA-seq Library Preparation

This protocol is built upon protocols from Kumar et al. 2012 (1) and from Wang et al. 2011 (2). This protocol starts with ground plant tissue, rather than purified RNA and lends itself to automation in a 96 well format.

Notes before beginning:

1. Before starting prep, pull out Lysis Binding Buffer Partial (LBBP), LIDS in the buffer will have precipitated if stored at 4°C.
2. It is very important to start with ≤100 mg of tissue or your starting lysate may be too viscous.
3. We grind the plant tissue in a Retsch Tissue Homogenizer in screw cap 2.0 ml tubes (Fisher Tubes cat. no. 02-682-558) with 3 stainless steel balls (3.2mm diameter, cat. no. 11079132ss). We use screw cap tubes to avoid popping of caps. We grind the leaf tissue until it is in a fine 'minty' green powder.
4. Prepare plenty of DEPC-treated water for making stock solutions and buffers and for cleaning scissors and forceps for tissue collection.
5. Work flow of the following mRNA protocol: grind plant tissue/homogenize in lysis buffer, bind sample to beads, wash, elute, DNase treat, heat treat, 2nd binding to beads, 2nd wash, 2nd elution.

Stocks needed (RNase Free):

1 M Tris-HCL, pH 7.5
8 M LiCL
3 M NaCl
0.5 M EDTA, pH 8

mRNA Extraction Buffers:

Lysis Binding Buffer (LBB)

LBB Partial (LBBP,can be made ahead of time and stored at 4°C)
100 mM Tris-HCl, pH 7.5
500 mM LiCl
10 mM EDTA, pH 8
1% LiDS (add directly)

Just before Homogenization add:
5 mM DTT (add directly)
2-Mercaptoethanol (5 µl/ml)
Antifoam A (5 µl/50 ml)

Mix thoroughly by vortexing until it is uniform.

Binding Buffer (BB)

20 mM Tris-HCl, pH 7.5
1.0 M LiCL
2 mM EDTA

Wash Buffer (WB)

10 mM Tris-HCl, pH 7.5
0.15 M LiCL
2 mM EDTA

EDTA Spike (ES):

0.5 M EDTA

Elution Buffer (EB)

10 mM Tris-HCL, pH 7.5

Other kits and reagents:

- Qubit RNA BR Assay Kit #Q10210 (Thermo Fisher Scientific, Waltham, MA)
- TURBO DNase #AM2238 (Thermo Fisher Scientific)
- Sera-Mag Beads #3815-2103-010150 **OR** Dynabeads Olgio (dT)25 #61002 (Thermo Fisher Scientific)

mRNA Preparation (~3.5 h for 12 samples, non-automated):

1.1. Grind Plant Tissue

1. We grind the plant tissue in a Retsch Tissue Homogenizer in screw cap 2.0 ml tubes (Fisher Tubes cat. no. 02-682-558) with 3 stainless steel balls (3.2mm diameter, cat. no. 11079132ss). We grind the leaf tissue until it is a fine 'minty' green powder. This requires between 3-4 45sec sets at 30.0Hz. Be sure to refreeze the samples in liquid nitrogen between grinding.

2. Add 1000 µl **LBB**/ 100 mg ground tissue. Immediately tap tube upside-down vigorously to get plant tissue into LBB. Vortex vigorously until all plant tissue is nicely in solution (not chunky).

3. Allow LBB+tissue mixture to sit at room temperature for 5 min. Transfer to a new tube to remove beads before spin.

4. Spin LBB+tissue in centrifuge at room temperature for 10 min at max speed.

5. Carefully move supernatant from cell debris pellet to a new tube (~600-800 µl lysate; Lo-bind DNA tube optional). **When pooling tissue from multiple plants, we pool the lysate at this step and store the remaining lysate at -80C for future RNA purification if needed. The tissue is much more stable in LBB.

----(This is a good pause point)

*****Important Note:** When performing all wash steps it is important to wash samples by very gently pipetting up and down. Pipette up and down until beads are resuspended (don't overdo it),

if beads are stuck to the wall of the tube a gentle scrubbing motion with the tip of the pipette is fine. Although we did not see any difference when vortexing to mix we prefer pipetting. Lo-Bind DNA tubes are not necessary for all steps but do make bead resuspension much easier.

1.2. Bind Sample to Beads

6. Wash Oligo (dt)25 beads twice with 120 µL of Binding Buffer (30 µL/sample of Fisher Sera-Mag Beads cat. no. 3815-2103-010150 **OR** 60 µL/sample of Life Technologies Dynabeads Olgio (dT)25 cat. no. 61002). Re-suspend washed Oligo (dt)25 beads in 50 µL/sample of Binding Buffer.

7. Heat **1000 µL LBB** lysate (LBB + Plant Tissue supernatant) at 65°C for 2 min (heat plate). Quick spin to bring down any liquid from the cap of the tube.

8. Place **50 µL of beads** /sample in a new 1.5 ml tube and remove binding buffer, immediately apply **1000 µL of heat-treated LBB tissue lysate** to the Oligo (dt)25 beads and shake samples on vortex for 15 min at setting of 1.5 at room temperature. Can quick spin in microfuge if mixture remains on walls of tube.

9. Put on magnetic stands (wait until the liquid is clear and beads are pulled to the side of the tube) and then remove liquid without disturbing beads. Be sure to check the pipette tip for any beads in the buffer before discarding. If beads are visible, return buffer to tube and wait 2 min for solution to clear. **We found that a homemade magnetic rack made of plexiglass and magnets purchased from www.kjmagnetics.com works best.

1.3. Wash Sample

10. Wash the beads+sample **twice** with **120 µL of WB**. After the application of the second wash a light spin with the picofuge is recommended so all wash buffer can be removed. Put on magnetic stands (wait until the liquid is clear and beads are pulled to the side of the tube) and then remove liquid without disturbing beads.

1.4. DNase Treatment

Note: Take Qubit Dye out of the refrigerator during the DNase Treatment but keep in the dark. Dye is in DMSO and needs time to thaw.

11. Add **44 µL of EB** and elute sample off beads at 80°C for 2min with shaking. Quick spin.

12. Place sample on magnetic rack, wait until liquid is clear then remove eluted sample and add to a new set of tubes containing **5 µL of Turbo DNAse buffer**. Add **1 µL of Turbo DNase**. Incubate for at 37°C for 30min. Quick spin.

13. Add **120 µL of RNase free H20** to beads. Wash **twice** with 120 µL of RNase free H20. Remove water and add **50 µL of BB** onto the beads to prime them for the second binding after DNase treatment. Mix by carefully pipetting between each step.

1.5. Heat treatment and 2nd Binding to Beads

14. Add **3.8 µL** of 0.5 M EDTA to each DNase treated sample.

15. Heat treat sample+EDTA at 75°C for 10 min (NO SHAKING). Quick spin.

16. Add sample back onto the beads in 50 µL of Binding Buffer (step 13). Shake on vortex at room temperature for 15 min, setting of 1.5. Quick spin.

Note: While sample is being applied back onto the beads take out the supplies necessary for the Bioanalyzer (used to QC mRNA). The reagents must sit at room temperature for 30 min.

1.6. 2nd Wash and Elution

17. Wash sample **twice** with **120 µL of WB.** After the application of the second wash a light spin with the picofuge is recommended so all wash buffer can be removed. Put on magnetic stands (wait until the liquid is clear and beads are pulled to the side of the tube) and then remove liquid without disturbing beads.

18. Add **50 µL of EB** and elute sample off beads at 80°C for 2min with shaking. Place sample on magnetic rack, wait until liquid is clear then remove eluted sample and add to a new set of tubes. Keep on ice while washing beads.

19. Add **120 µL of RNase free H20** to beads. Wash **twice** with 120 µL of RNase free H20. Remove water and add **50 µL of BB** onto the beads to prime them for binding. Mix by carefully pipetting between each step.

20. Add sample back onto the beads in 50 µL BB. Shake on vortex at room temperature for 15 min, setting of 1.5. Quick spin.

1.7. 3rd Wash and Elution

21. Wash sample **twice** with **120 µL of WB.** After the application of the second wash a light spin with the picofuge is recommended so all wash buffer can be removed. Put on magnetic stands (wait until the liquid is clear and beads are pulled to the side of the tube) and then remove liquid without disturbing beads.

22. Add **16 µL of EB** and elute sample off beads at 80°C for 2min with shaking. Quick spin. Place sample on magnetic rack, wait until liquid is clear then remove eluted sample and add to a new set of tubes.

Quality Control:

1. Take **2 µL of eluted sample** for quantification with qubit.
2. After quantification with qubit check quality of mRNA on bioanalyzer using a Plant RNA PICO chip (dilute aliquot of sample if necessary to ~2.5ng/µL). **We typically get concentrations of ~30ng/µL on the Qubit.

Notes/Comments

While developing the above protocol, several variations were tested to optimize the efficiency and quality of the mRNA. For completeness, we have described these variations below as well as

the bioanalyzer results from our failed attempts. We hope this information will help users troubleshoot any problems they face.

2nd Heat Treatment

- We found a significant reduction in rRNA contamination with the addition of the heat treatment at step 16 (bioanalyzer electropherograms sample 1 vs. 2).

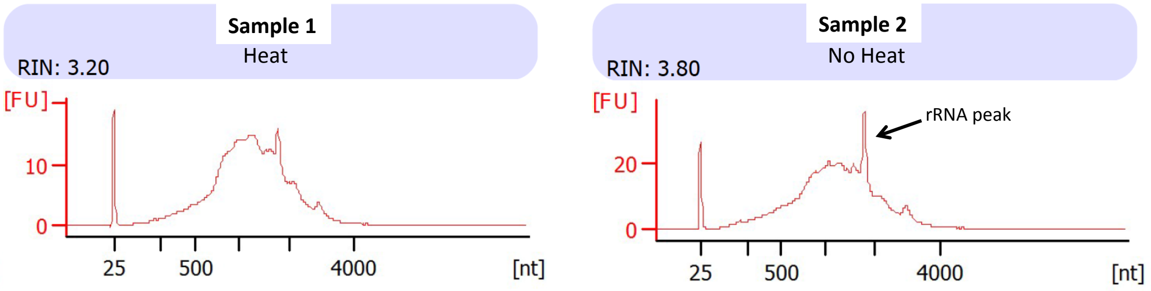

Extraction of total RNA from tissue lysate in LBB

Stocks needed (RNase Free):

1 M Tris-HCL, pH 7.5
10 M LiCL
3 M NaCl
5M NH_4OAC
DEPC-treated H_2O
PCI pH 4.5 (Can also make a PC with equal parts phenol pH4.5 + chloroform)
70% ETOH

Resuspension Solution (RS):

50mM Tris-Cl (pH 7.5)
50mM NaCl

1. Add 800 µL LiCl and 16 µL 2-Mercaptoethanol to 200 µL RNA lysate for a final concentration of 8M LiCl/2% 2-Mercaptoethanol. Mix well and leave on ice at 4°C overnight.
2. Spin down lysate for 30 min max speed at 4°C**.**
3. Pipette off supernatant, expect large lipid blobs.
4. Add 1mL 70% ETOH to pellet, mix well to wash walls of tube.
5. Spin max speed for 10 min at 4°C.
6. Pour off ETOH and air dry pellet, takes some time, can put in front of a fan if needed. Do not leave pellets sitting dry.
7. Resuspend pellet in 100 µL RS.
8. Add 100 µL PCI or PC and vortex for 20 sec to mix.
9. Spin max speed for 5 min at 4°C.
10. Transfer aqueous phase (~100 µL) to a new tube and add 1 volume of chloroform. Vortex for 20 sec to mix and spin max speed for 5 min at 4°C.

11. Transfer aqueous layer (~80 µL) to new tube and add 50 µL 5M NH_4OAC and mix. Add 325 µL 100% ETOH and mix. Put in -80°C for ~4hrs or until sample becomes thick. *****Possible stop point. Sample can be left overnight*****
12. Spin 20 min max speed at 4°C.
13. Pour off supernatant and invert tubes on KimWipes for a few minutes (~10 min) to drain ethanol from pellet. Let air dry.
14. Add 30 µL DEPC-treated water to pellet and incubate on ice to resuspend, may take up to 1hr. Any insoluble material should be pelleted and avoided.
15. Measure concentration on NanoDrop.

References

1 - Kumar, R., Ichihashi, Y., Kimura, S., Chitwood, D.H., Headland, L.R., Peng, J., Maloof, J.N., and Sinha, N.R. (2012). A High-Throughput Method for Illumina RNA-Seq Library Preparation. Front. Plant Sci. **3**: 202. doi: 10.3389/fpls.2012.00202

2 - Wang, L., Si, Y., Dedow, L.K., Shao, Y., Liu, P., and Brutnell, T.P. (2011a). A low-cost library construction protocol and data analysis pipeline for Illumina-based strand-specific multiplex RNA-seq. PLoS One **6**: e26426. Doi: 10.1371/journal.pone.0026426
