# Supplementary material for: Temporal network analysis identifies early physiological and transcriptomic indicators of mild drought in Brassica rapa
Source: eLife. 2017 Aug 18;6:e29655. doi: 10.7554/eLife.29655 (PMC5628015; doi:10.7554/eLife.29655)
Supplement: Transparent reporting form [file elife-29655-transrepform.docx]

***eLife’s* transparent reporting form**

We encourage authors to provide detailed information *within their submission* to facilitate the interpretation and replication of experiments. If you have any questions, please contact us: [editorial@elifesciences.org](mailto:editorial@elifesciences.org).

**Sample-size estimation**

- You should state whether an appropriate sample size was computed when the study was being designed
- You should state the statistical method of sample size computation and any required assumptions
- If no explicit power analysis was used, you should describe how you decided what sample (replicate) size (number) to use

Please outline where this information can be found within the submission (e.g., page numbers or figure legends), or explain why this information doesn’t apply to your submission:

1. Physiology – All sample sizes are reported in the legends of Figure1, 2 & 3. Sample size was based on possible measurements/harvestings in 30 minutes to minimize the noise with the tissue collection for RNA-seq.

2. RNA-seq sample size indicated on p.20. The sample size was based on the cost associated with sequencing over a 2-day time course. To have 4h sampling density we were limited to 2 biological replicates.

3. NanoString sample size indicated on p. 24.

**Replicates**

- You should report how often each experiment was performed
- You should include a definition of biological versus technical replication
- The data obtained should be provided and sufficient information should be provided to indicate the number of independent biological and/or technical replicates
- If you encountered any outliers, you should describe how these were handled
- Criteria for exclusion/inclusion of data should be clearly stated
- High-throughput sequence data should be uploaded before submission, with a private link for reviewers provided (these are available from both GEO and ArrayExpress)

Please outline where this information can be found within the submission (e.g., page numbers or figure legends), or explain why this information doesn’t apply to your submission:

1. Physiology Experiment Replication - Soil moisture (*n*=6 to 20), Gas exchange (*n*=6 to 12), Chl *a,* fluorescence (*n*=6 to 12) every 4 h over 2-days. Sugar analysis on flash frozen samples (*n*= 8 to 12) every 4 h except for ZT9 on both days. Above-ground biomass of 12 samples at ZT9 on both Day1 and Day2. Detailed description pp. 19-21.

2. RNA-seq - p. 20 and detailed description in the GEO submission GSE90841.

3. NanoString - pp.24-25

**Statistical reporting**

- Statistical analysis methods should be described and justified
- Raw data should be presented in figures whenever informative to do so (typically when N per group is less than 10)
- For each experiment, you should identify the statistical tests used, exact values of N, definitions of center, methods of multiple test correction, and dispersion and precision measures (e.g., mean, median, SD, SEM, confidence intervals; and, for the major substantive results, a measure of effect size (e.g., Pearson's r, Cohen's d)
- Report exact p-values wherever possible alongside the summary statistics and 95% confidence intervals. These should be reported for all key questions and not only when the p-value is less than 0.05.

1. Physiology – p.22 includes a description of statistical analyses utilized to analyze the physiology data.

2. NanoString – pp.24-25 includes a description of the tests performed to detect outlier probes and to calculate significant difference between treatments.

Please outline where this information can be found within the submission (e.g., page numbers or figure legends), or explain why this information doesn’t apply to your submission:

(For large datasets, or papers with a very large number of statistical tests, you may upload a single table file with tests, Ns, etc., with reference to page numbers in the manuscript.)

**Additional data files (“source data”)**

- We encourage you to upload relevant additional data files, such as numerical data that are represented as a graph in a figure, or as a summary table
- Where provided, these should be in the most useful format, and they can be uploaded as “Source data” files linked to a main figure or table
- Include model definition files including the full list of parameters used
- Include code used for data analysis (e.g., R, MatLab)
- Avoid stating that data files are “available upon request”

Please indicate the figures or tables for which source data files have been provided:

Figure 1 – Source data 1 contains chamber conditions for the experiment and the biomass and Fv’/Fm’ results for the two replicate experiments.

Figure 3 – Source data 1 contains mean values for the physiology measurements.

Figures 4&7 – Supplementary File 1 contains the gene lists for the networks with their corresponding module assignment, module membership and associated p. value as well as their gene significance values with the physiology traits.

Figures 8&9 – Supplementary File 2 contains the JTK-Cycle analysis results used for identifying drought responsive genes.

Figure 10 – Source data 1 contains the list of NanoString target genes.
