# Supplementary material for: Temporal network analysis identifies early physiological and transcriptomic indicators of mild drought in Brassica rapa
Source: eLife. 2017 Aug 18;6:e29655. doi: 10.7554/eLife.29655 (PMC5628015; doi:10.7554/eLife.29655)
Supplement: Peer review history

# Decision letter

**DOI:** 10.7554/eLife.29655.025

**Affiliations:** University of British ColumbiaCanada

In the interests of transparency, eLife includes the editorial decision letter and accompanying author responses. A lightly edited version of the letter sent to the authors after peer review is shown, indicating the most substantive concerns; minor comments are not usually included.

Thank you for submitting your article "Temporal network analysis identifies early physiological and transcriptomic indicators of mild drought in *Brassica rapa*" for consideration by *eLife*.

Your article has been reviewed by two peer reviewers, and the evaluation has been overseen by Joerg Bohlmann as the Reviewing Editor and Christian Hardtke as the Senior Editor. The two external reviewers, whose comments are following below, were very positive. The Reviewing Editor agrees with this positive assessment, and he is recommending your paper to be accepted for publication in *eLife* pending revisions to address the reviewers' comments.

Reviewer #1:

In this manuscript, the authors investigate the early drought response of B. rapa. They identify "mild drought" conditions where B. rapa plants begin to react to drought without overt symptoms. Under mild drought and well-watered conditions, the authors measure diel patterns of gene expression and assess temporal changes in physiological readouts known to be influenced by water deficit and/or part of drought responses. The authors identify co-expression modules with WGCNA network analysis. They then correlate these modules with observed physiological changes occurring under drought conditions. With a unique circadian-guided filter approach, the authors identify regulatory modules associated with time of day aspects of drought responses. This approach identified several modules where gene expression of module members is highly correlated with at least one of the phenotypic traits. While these correlations do not provide specific regulatory connections within drought response networks, the study identifies several modules wherein genes are related by function and these functions are plausible contributors to drought responses.

There are no substantive concerns with this manuscript. The data are solid, with the caveat that the network analysis is outside my expertise. The text is well written and logically organized. This is an important paper because it shows the significance of temporal dynamics in drought response and, more generally, that consideration of time of day is critical when investigating plant stress responses. It also lays out relationships between genes that together are highly likely to shape the drought response of B. rapa.

Reviewer #2:

The study by Greenham and colleagues aims to develop an integrated view of mild drought stress response in plants. Using a single genotype of Brassica rapa as their model, the authors performed a soil dry down experiment and measured ecophysiological parameters and transcript abundance (RNASeq) in a two-day diel framework. They perform quite a few analyses on these data, both separately and together, and identify some very interesting patterns in the data.

On the whole, this is a truly exceptional experiment and will be among the very best studies of response to soil drying in plants. The experiment itself and sampling represents a tremendous amount of work, and was executed at the very highest standards of stress implementation, experimental control, and empirical measurements. It is rare to see (relevant) high-quality ecophysiological measurements and RNASeq measurements in a single experiment, and the field needs many more studies of this type. I anticipate that other researchers may utilize this dataset and yield address additional, interesting, insights beyond those explored here.

I have several suggestions for improving the manuscript, which I enumerate below.

1) A major advancement of this study is the ability to integrate ecophysiology parameters with transcript information, using temporal variation to assess formal correlations. It is unclear how these correlations were made. In subsection “Correlating network modules with phenotypic traits”: what physiology values were used for these correlations? As written, it sounds as if the correlation matrix is just all of the modules against means of all of the ecophys parameters(?). And then visual inspection was made to assess the extent to which the diurnal patterns match between the two types of data? This non-quantitative analysis continues in the following section, where e.g. subsection “Applying a circadian-guided approach to identify drought responsive genes” states that two dry modules "match the pattern observed in the gs data." At the very least, please explain in more detail how these analyses were performed. But I also wonder if a more formal analysis of the diurnal patterns might be fruitful, incorporating the full–time series for each type of data?

2) More formal language describing variance, correlation, and statistical significance should be used. "Varying degrees of significance" subsection “Correlating network modules with phenotypic traits”, and "high significance measures" imply that a statistical test with a p-value of 0.001 is somehow MORE significant than a test with a p-value of 0.01; this is not the case. Both tests are significant provided that they fall below some a priori value of α. Please remove these statements and just state that the tests are significant. In subsection “Applying a circadian-guided approach to identify drought responsive genes”, a claim is made about one correlation being higher than another. Contrasting the strength of two correlations requires a formal test but, frankly, doing such tests is unnecessary for this study's aims and conclusions; I suggest simply removing these qualitative statements about differing strengths of correlation.

3) Subsection “Applying a circadian-guided approach to identify drought responsive genes”, as presented in Figure 8B, top panel, does not support the statement that the dM1 module is enriched for "macromolecule metabolic processes"

4) Figure 9 is confusing. First, the color key is included in panel C, but (I think) is also valid for panel B. Second, there is no description of panel C in the figure legend.

5) The inclusion of the NanoString analysis to support conclusions from the RNASeq data is great. I'm not sure how the types of data actually support each other, however. For example, subsection “Applying a circadian-guided approach to identify drought responsive genes” states "the diel expression patterns seen.… were recapitulated for the genes evaluated (Figure 10)." What are readers meant to assess in Figure 10? That the genes are cycling? Given that we have not seen the circadian patterns of these specific genes in the RNASeq data, how are we to determine whether there is concordance between the data sets? As written, this whole section supports the concordance between these NanoString-measured genes and PAST research, but there is no real validation offered between the NanoString and RNASeq datasets. Perhaps a few sentences could be changed so that the point of this paragraph is to validate the RNA samples themselves in light of past work, rather than formally validate the RNASeq results.

# Reply: Author response

**DOI:** 10.7554/eLife.29655.026

*Reviewer #1:*

*In this manuscript, the authors investigate the early drought response of B. rapa. They identify "mild drought" conditions where B. rapa plants begin to react to drought without overt symptoms. Under mild drought and well-watered conditions, the authors measure diel patterns of gene expression and assess temporal changes in physiological readouts known to be influenced by water deficit and/or part of drought responses. The authors identify co-expression modules with WGCNA network analysis. They then correlate these modules with observed physiological changes occurring under drought conditions. With a unique circadian-guided filter approach, the authors identify regulatory modules associated with time of day aspects of drought responses. This approach identified several modules where gene expression of module members is highly correlated with at least one of the phenotypic traits. While these correlations do not provide specific regulatory connections within drought response networks, the study identifies several modules wherein genes are related by function and these functions are plausible contributors to drought responses.*

*There are no substantive concerns with this manuscript. The data are solid, with the caveat that the network analysis is outside my expertise. The text is well written and logically organized. This is an important paper because it shows the significance of temporal dynamics in drought response and, more generally, that consideration of time of day is critical when investigating plant stress responses. It also lays out relationships between genes that together are highly likely to shape the drought response of B. rapa.*

We thank the reviewer for their positive comments. We agree that the network analysis only provides correlations that are helpful for generating further hypotheses about drought response. We are testing these in ongoing work to assess specific regulations and interactions of the modules and physiology traits.

*Reviewer #2:*

*The study by Greenham and colleagues aims to develop an integrated view of mild drought stress response in plants. Using a single genotype of Brassica rapa as their model, the authors performed a soil dry down experiment and measured ecophysiological parameters and transcript abundance (RNASeq) in a two-day diel framework. They perform quite a few analyses on these data, both separately and together, and identify some very interesting patterns in the data.*

*On the whole, this is a truly exceptional experiment and will be among the very best studies of response to soil drying in plants. The experiment itself and sampling represents a tremendous amount of work, and was executed at the very highest standards of stress implementation, experimental control, and empirical measurements. It is rare to see (relevant) high-quality ecophysiological measurements and RNASeq measurements in a single experiment, and the field needs many more studies of this type. I anticipate that other researchers may utilize this dataset and yield address additional, interesting, insights beyond those explored here.*

We thank the reviewer for the thoughtful comments and suggestions that have improved the clarity of the manuscript.

*1) A major advancement of this study is the ability to integrate ecophysiology parameters with transcript information, using temporal variation to assess formal correlations. It is unclear how these correlations were made. In subsection “Correlating network modules with phenotypic traits”: what physiology values were used for these correlations? As written, it sounds as if the correlation matrix is just all of the modules against means of all of the ecophys parameters(?). And then visual inspection was made to assess the extent to which the diurnal patterns match between the two types of data? This non-quantitative analysis continues in the following section, where e.g. subsection “Applying a circadian-guided approach to identify drought responsive genes” states that two dry modules "match the pattern observed in the gs data." At the very least, please explain in more detail how these analyses were performed. But I also wonder if a more formal analysis of the diurnal patterns might be fruitful, incorporating the full–time series for each type of data?*

We have clarified the explanation of the module – trait correlations in subsection “Correlating network modules with phenotypic traits “as well including a more detailed description in the methods section under WGCNA network analysis (subsection “NanoString sample preparation and analysis”). Briefly, correlations were conducted for each physiology trait separately using the module eigengene and the mean values of the physiology measurements at each time point. To assess the extent to which the diurnal patterns match we used the results from these correlations shown in Figure 7 where positive correlations are shown in red. We revised subsection “Applying a circadian-guided approach to identify drought responsive genes” to clarify that “dM1 and dM5 genes exhibit peak expression levels at ZT5 as do the *g*_s_ data, consistent with the positive correlation of these modules with *g*_s_.”

*2) More formal language describing variance, correlation, and statistical significance should be used. "Varying degrees of significance" subsection “Correlating network modules with phenotypic traits”, and "high significance measures" imply that a statistical test with a p-value of 0.001 is somehow MORE significant than a test with a p-value of 0.01; this is not the case. Both tests are significant provided that they fall below some a priori value of α. Please remove these statements and just state that the tests are significant. In subsection “Applying a circadian-guided approach to identify drought responsive genes”, a claim is made about one correlation being higher than another. Contrasting the strength of two correlations requires a formal test but, frankly, doing such tests is unnecessary for this study's aims and conclusions; I suggest simply removing these qualitative statements about differing strengths of correlation.*

We have removed the qualitative statements and revised the statements of significance. We have also clarified the ‘high significance measures’ statement (subsection “Applying a circadian-guided approach to identify drought responsive genes”) that is describing the Gene Significance measure that is a measure of the correlation between the gene expression values at each time point with the physiology data at each time point.

*3) Subsection “Applying a circadian-guided approach to identify drought responsive genes”, as presented in Figure 8B, top panel, does not support the statement that the dM1 module is enriched for "macromolecule metabolic processes"*

Thank you for pointing this out. We have corrected that line of text in Subsection “Applying a circadian-guided approach to identify drought responsive genes” to read ‘response to abiotic stimulus’.

*4) Figure 9 is confusing. First, the color key is included in panel C, but (I think) is also valid for panel B. Second, there is no description of panel C in the figure legend.*

We apologize for the confusion and have corrected the legends for Figures 8 and 9.

*5) The inclusion of the NanoString analysis to support conclusions from the RNASeq data is great. I'm not sure how the types of data actually support each other, however. For example, subsection “Applying a circadian-guided approach to identify drought responsive genes” states "the diel expression patterns seen.… were recapitulated for the genes evaluated (Figure 10)." What are readers meant to assess in Figure 10? That the genes are cycling? Given that we have not seen the circadian patterns of these specific genes in the RNASeq data, how are we to determine whether there is concordance between the data sets? As written, this whole section supports the concordance between these NanoString-measured genes and PAST research, but there is no real validation offered between the NanoString and RNASeq datasets. Perhaps a few sentences could be changed so that the point of this paragraph is to validate the RNA samples themselves in light of past work, rather than formally validate the RNASeq results.*

We have included a Figure 10—figure supplement 2 with the RNAseq expression data for the genes quantified using the Nanostring analysis in Figure 10 to show the consistent diel expression patterns mentioned in the text. We have also circled the nodes in Figures 8C and 9C that correspond to the genes analyzed by Nanostring to allow readers to compare the Nanostring expression patterns with the mean expression plots for the modules shown in Figures 8A and 9A. While we did mention past research as support for the role of these genes in drought response we also emphasize the specific time of day responses that we observed in our network analysis and validated using the Nanostring.
